# Supplementary material for: Current and future scenarios of suitability and expansion of cassava brown streak disease, Bemisia tabaci species complex, and cassava planting in Africa
Source: PeerJ. 2024 May 31;12:e17386. doi: 10.7717/peerj.17386 (PMC11146326; doi:10.7717/peerj.17386)
Supplement: Supplemental Information 1 — Model performance (Fig. A1), uncertainty prediction (Figs. B1 & B2), and the prediction of the current and future distribution of cassava, whitefly and CBSD. [file peerj-12-17386-s001.docx]

**Supplementary materials**

**Current and future scenarios of suitability and expansion of cassava brown streak disease, *Bemisia tabaci* species complex, and cassava planting in Africa**

Geofrey Sikazwe^1,2,3^, Rosita E. E. Yocgo^2,4^, Pietro Landi^1,5^, David M. Richardson^6^, Cang Hui^1,5,7^

^1^Department of Mathematical Sciences, Stellenbosch University, Stellenbosch 7602, South Africa; ^2^African Institute for Mathematical Sciences, Kigali, Rwanda; ^3^Mkwawa University College of Education, P.O. Box 2513, Iringa, Tanzania. ^4^Institute for Plant Biotechnology, Stellenbosch University, Stellenbosch 7602, South Africa; ^5^National Institute for Theoretical and Computational Sciences, Stellenbosch University, Stellenbosch 7602, South Africa; ^6^Centre for Invasion Biology, Department of Botany and Zoology, Stellenbosch University, Stellenbosch 7602, South Africa; ^7^Mathematical Bioscience Unit, African Institute for Mathematical Sciences, Cape Town 7945, South Africa

Correspondence: geofrey@aims.ac.za

This supplementary document contains supporting information for model performance presented as ROC curves (**Fig. A1**) and uncertainty in predictive capacity of twelve SDMs (**Fig.** **B1 & B2**). It also contains information on the prediction of the current distribution for whitefly using bioclimatic variables and cassava harvested area (**Fig. B3 A & B**), prediction of the current distribution for cassava brown streak disease using bioclimatic variables and cassava harvested area (**Fig. B3 C & D**).

Moreover, the supplementary material presents information on the projection of the current and future cassava suitable habitats in Africa (**Fig. C1 – C8**), projection of the current and future habitats at risk of whitefly, *Bemisia tabaci* invasion in Africa using (**Fig. D1 – D8**) and projection of the current and future habitats at risk of cassava brown streak invasion in Africa (**Fig. E1 – E8**). The future distribution of each species was projected using seven GCMs (**Table A1**).

| Model | Full name | Institute | Country | Reference |
| --- | --- | --- | --- | --- |
| BCC-CSM2-MR | Beijing Climate Center Climate System  Model | Beijing Climate Center | China | (Wu et al., 2019) |
| CNRM-CM6-1 | National Center for Meteorological  Research Earth System Model version 6 | National Center for Meteorological Research | France | (Voldoire et al., 2019) |
| CNRM-ESM2-1 | National Center for Meteorological  Research Earth System Model version 2 | National Center for Meteorological Research | France | (Séférian et al., 2019) |
| CanESM5 | The Canadian Earth System Model version 5 | Canadian Centre for Climate Modelling | Canada | (Swart et al., 2019) |
| IPSL-CM6A-LR | Institut Pierre-Simon Laplace Climete Model | Institut Pierre-Simon Laplace | France | (Bonnet et al., 2021) |
| MIROC-ES2L | Model for Interdisciplinary Research  on Climate, Earth System version 2 | Japan Agency for Marine-Earth Science  and Technology | Japan | (Hajima et al., 2019) |
| MIROC6 | Model for Interdisciplinary Research  on Climate version 6 | Japan Agency for Marine-Earth Science  and Technology | Japan | (Tatebe et al., 2019) |
| MRI-ESM2-0 | The Meteorological Research Institute  Earth System Model Version 2 | Meteorological Research Institute | Japan | (Yukimoto et al., 2019) |

**Appendix A:** List of Global Circulation Climate Models

**Table A1:** List of Global Circulation Climate Models (GCMs) used in the prediction the potential future distribution of Cassava, Whitefly (*Bemisia tabaci*) and Cassava brown streak disease in Africa.

**Appendix B:**  Supplementary figures


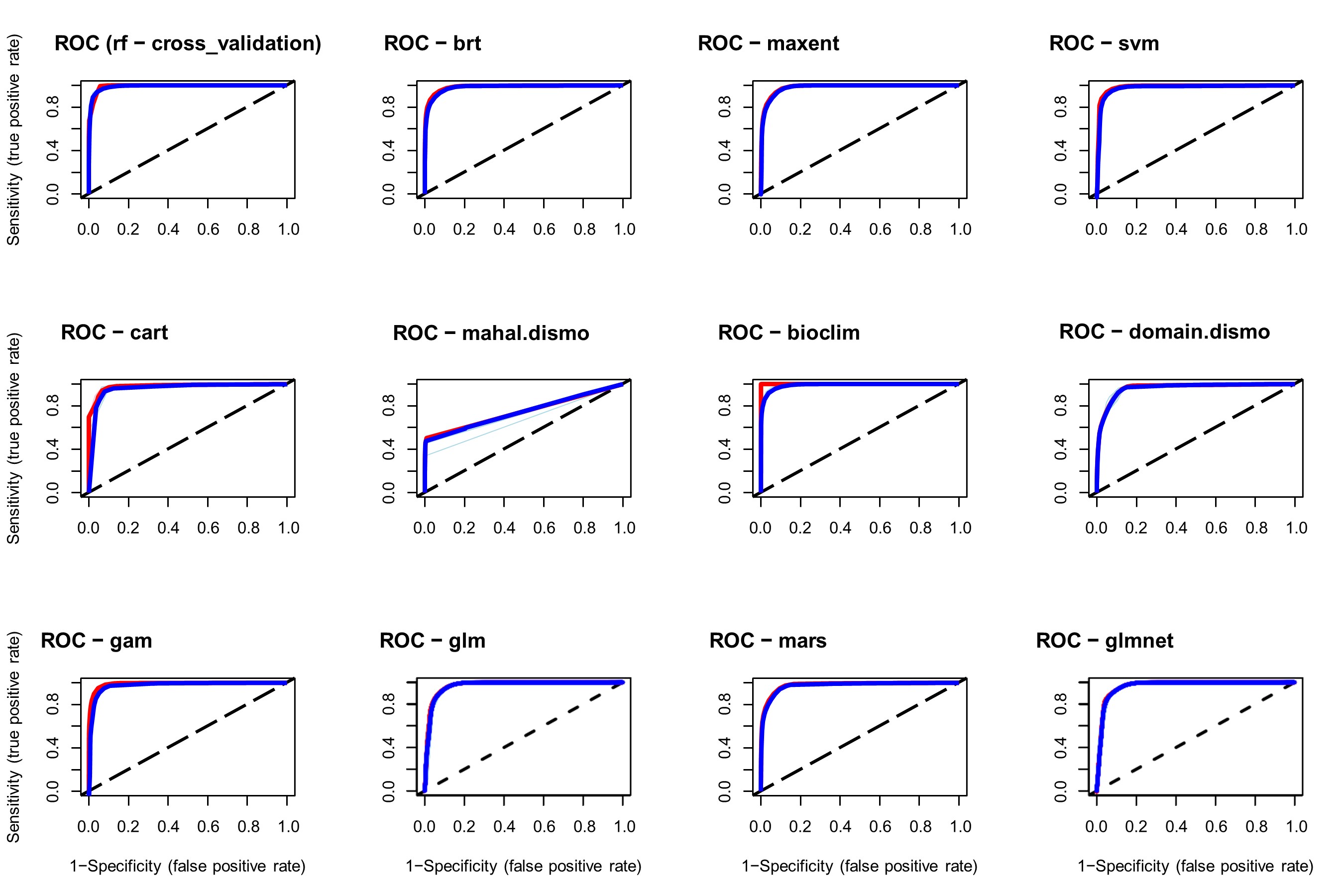


**Figure B1.** Receiver operating characteristic curve and AUC value under the current period (1970 - 2000) for cassava occurrence data.

*
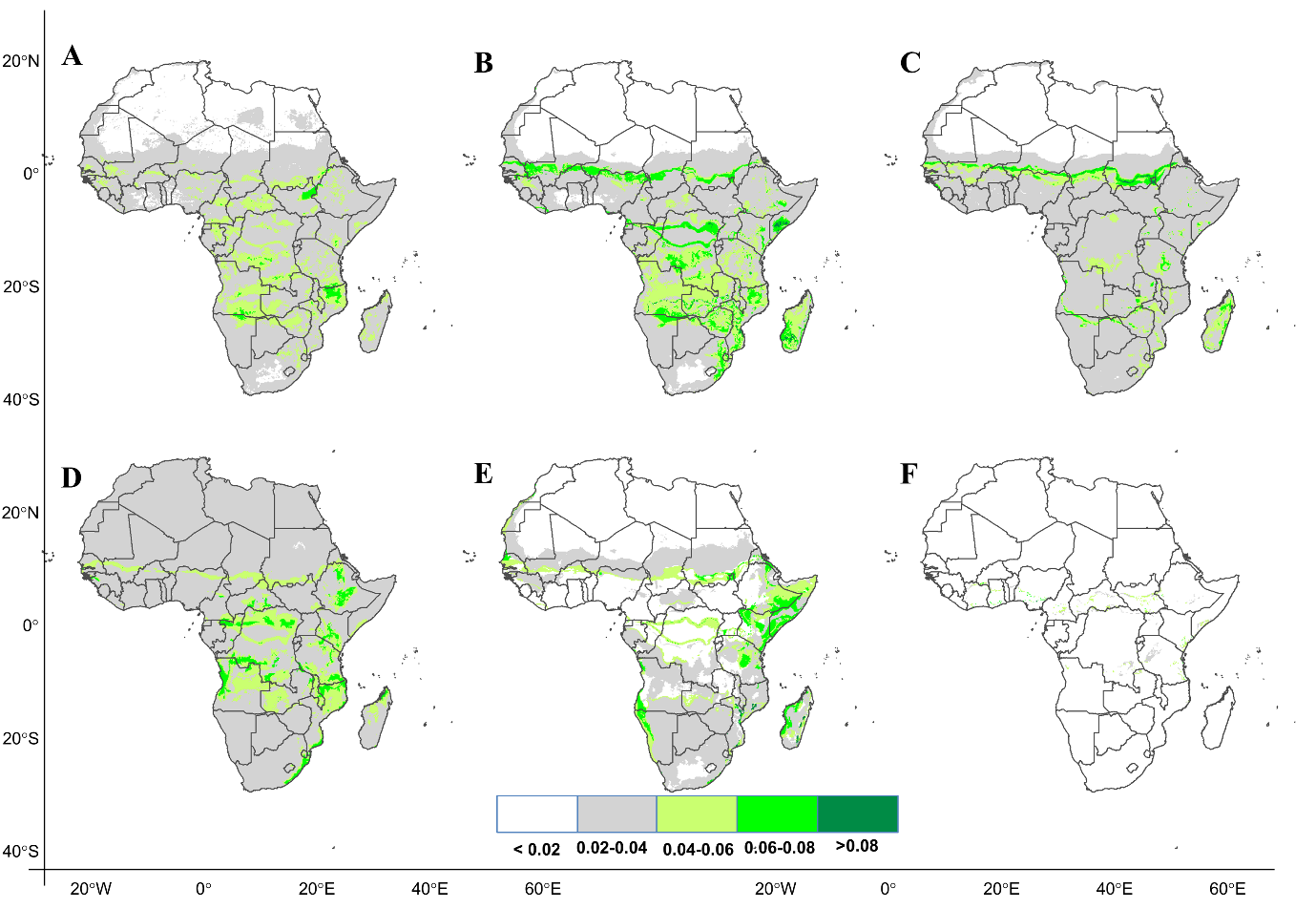
*

**Figure B2**. Comparison of predictive capacity of six models using cassava occurrence records. A) rf B) brt C) maxent D) svm E) cart and F) mahal.


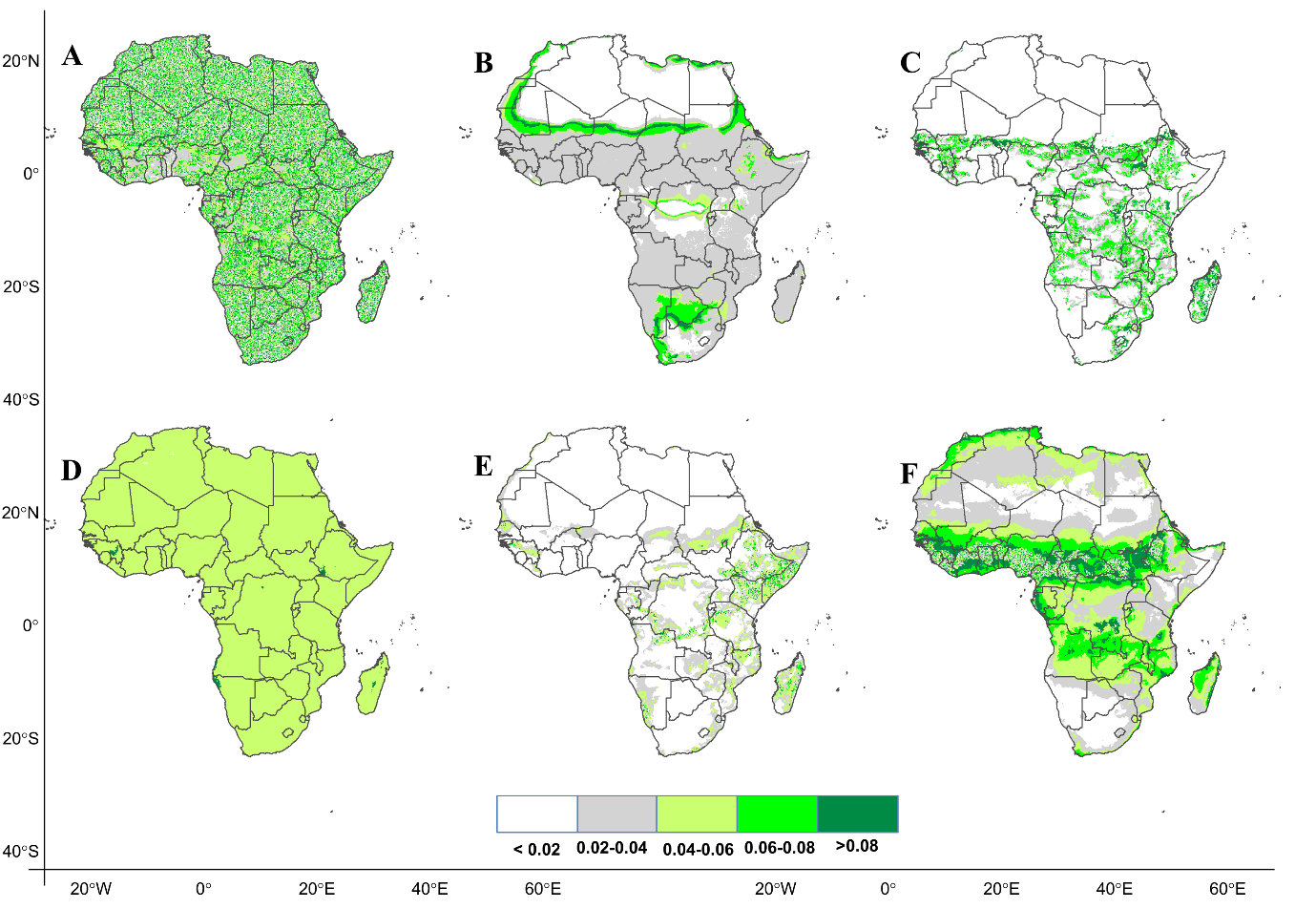


**Figure B3.** Comparison of predictive capacity of six models using cassava occurrence records. A) bioclim B) domain C) gam D) glm E) mars and F) glmnet.


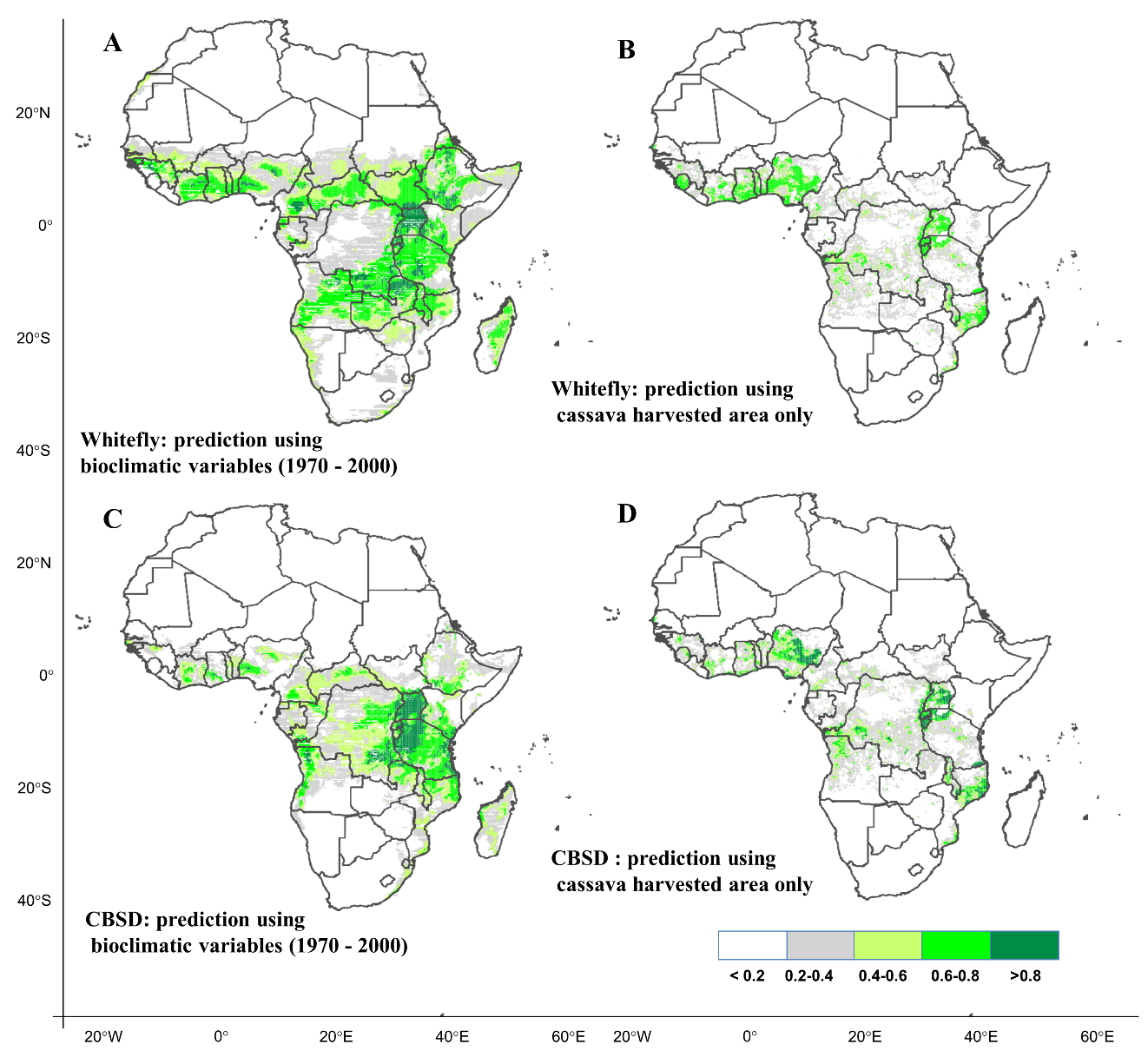


**Figure B4**. Predicted current distribution of whitefly, *B. tabaci* using A) bioclimatic variables B) using cassava harvested area*.* Predicted current distribution of cassava brown streak disease using C) bioclimatic variables and D) cassava harvested area and cassava suitability map.

**Appendix C:** Projection of the current and future cassava suitable habitats in Africa using eight GCMs***.***

***
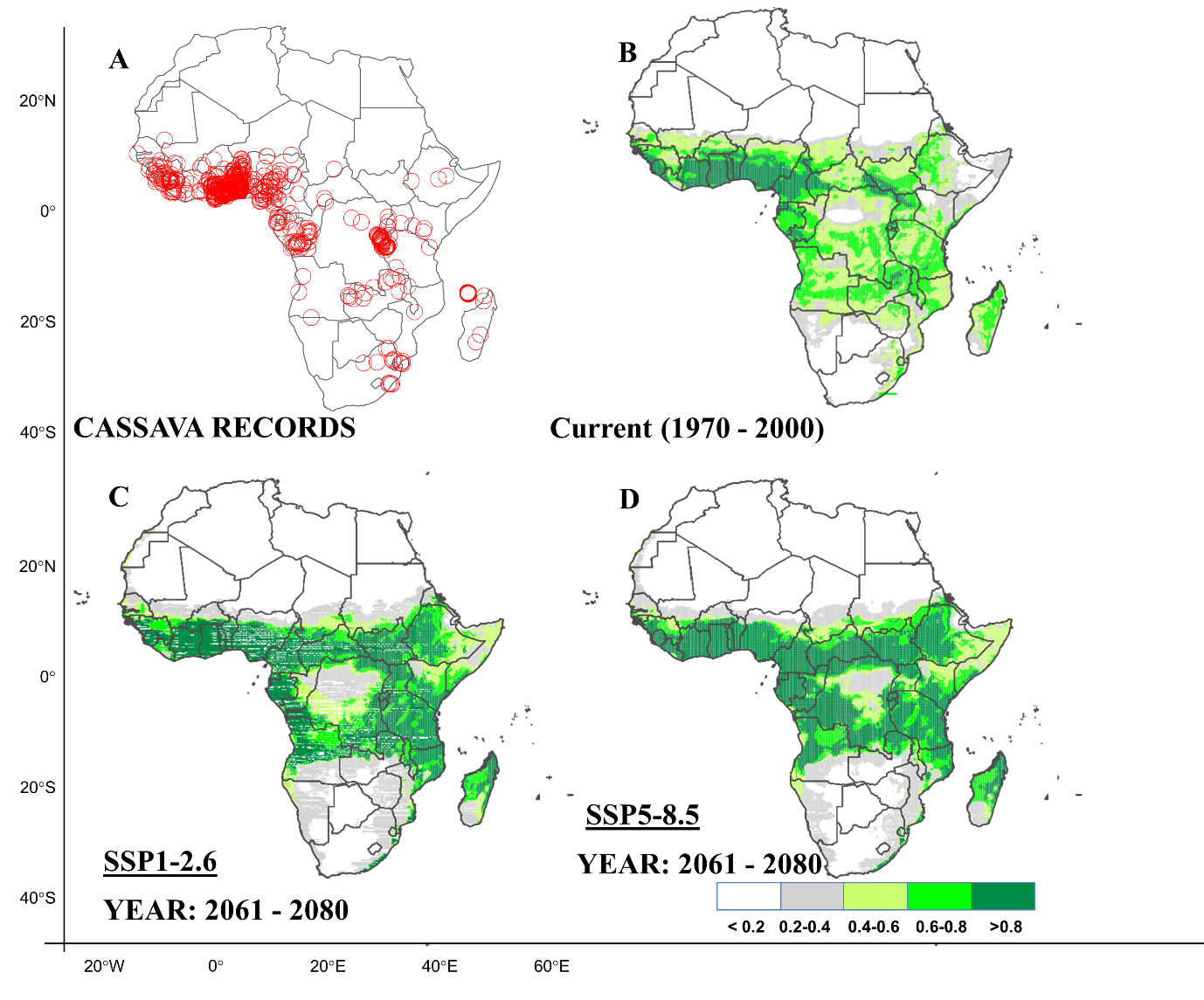
***

**Figure C1:** Current and future suitable habitats for cassava in Africa. Plots showing A) geolocations of cassava occurrence records provided by the global biodiversity information facility (GIBF; <https://www.gbif.org>), B) the predicted distribution for cassava under the current climate (1970-2000). Predicted future suitable habitats for cassava under C) the SSP1-2.6 scenario and D) the SSP5-8.5 scenario, using the **BCC-CSM2-MR** model for the year 2070s.


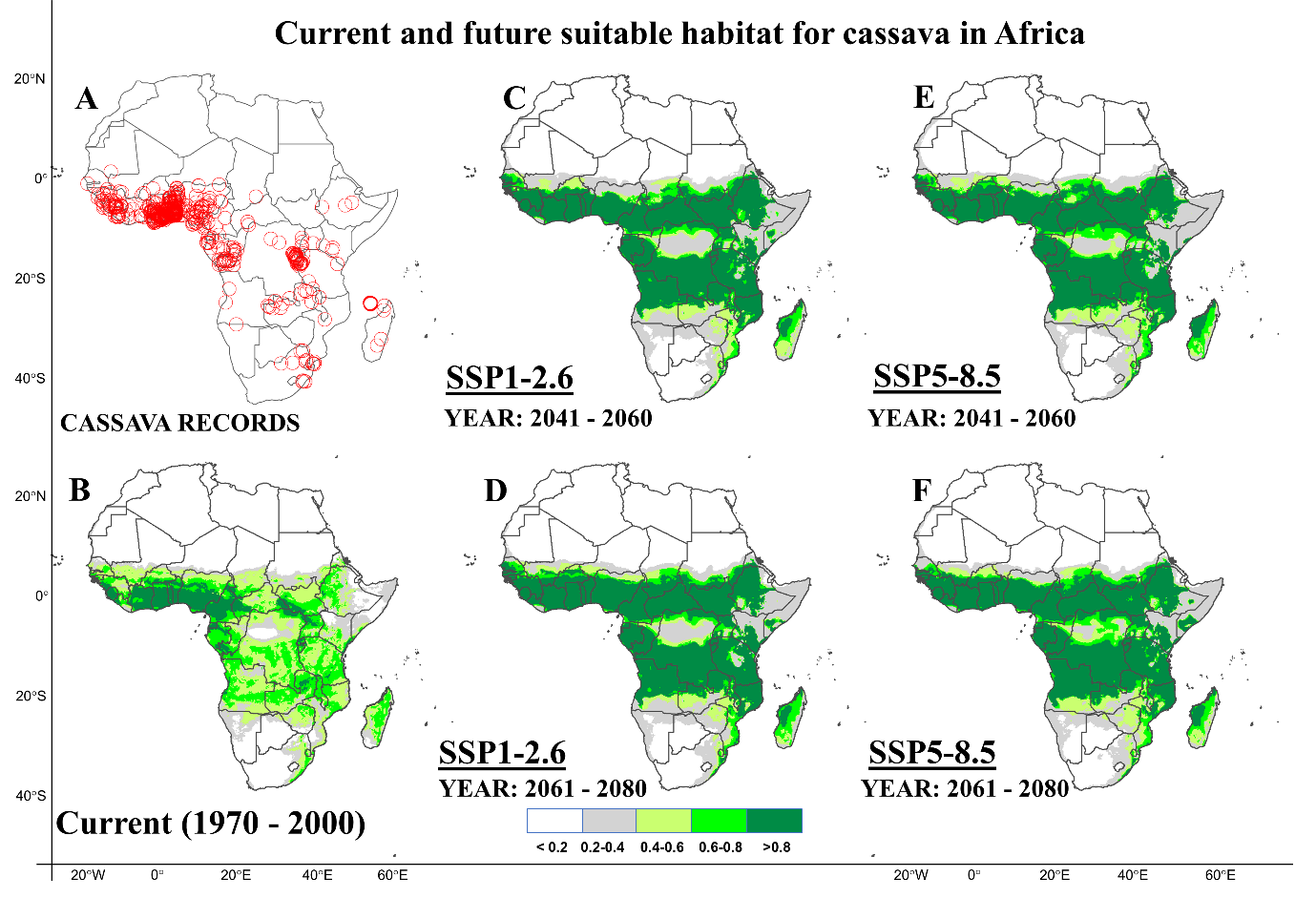


**Figure C2:** Current and future suitable habitats for cassava in Africa. Maps showing A) geolocations of cassava occurrence records provided by the global biodiversity information facility (GIBF; https://www.gbif.org), B) predicted spatial distribution of cassava under historical climate conditions (1970 - 2000). Future prediction of the suitable habitat under two shared social-economic pathways C) SSP1-2.6 (2050s), D) SSP1-2.6 (2070s), D) SSP5-8.5 and E) SSP5-8.5 using the **MRI-ESM2-0** model.


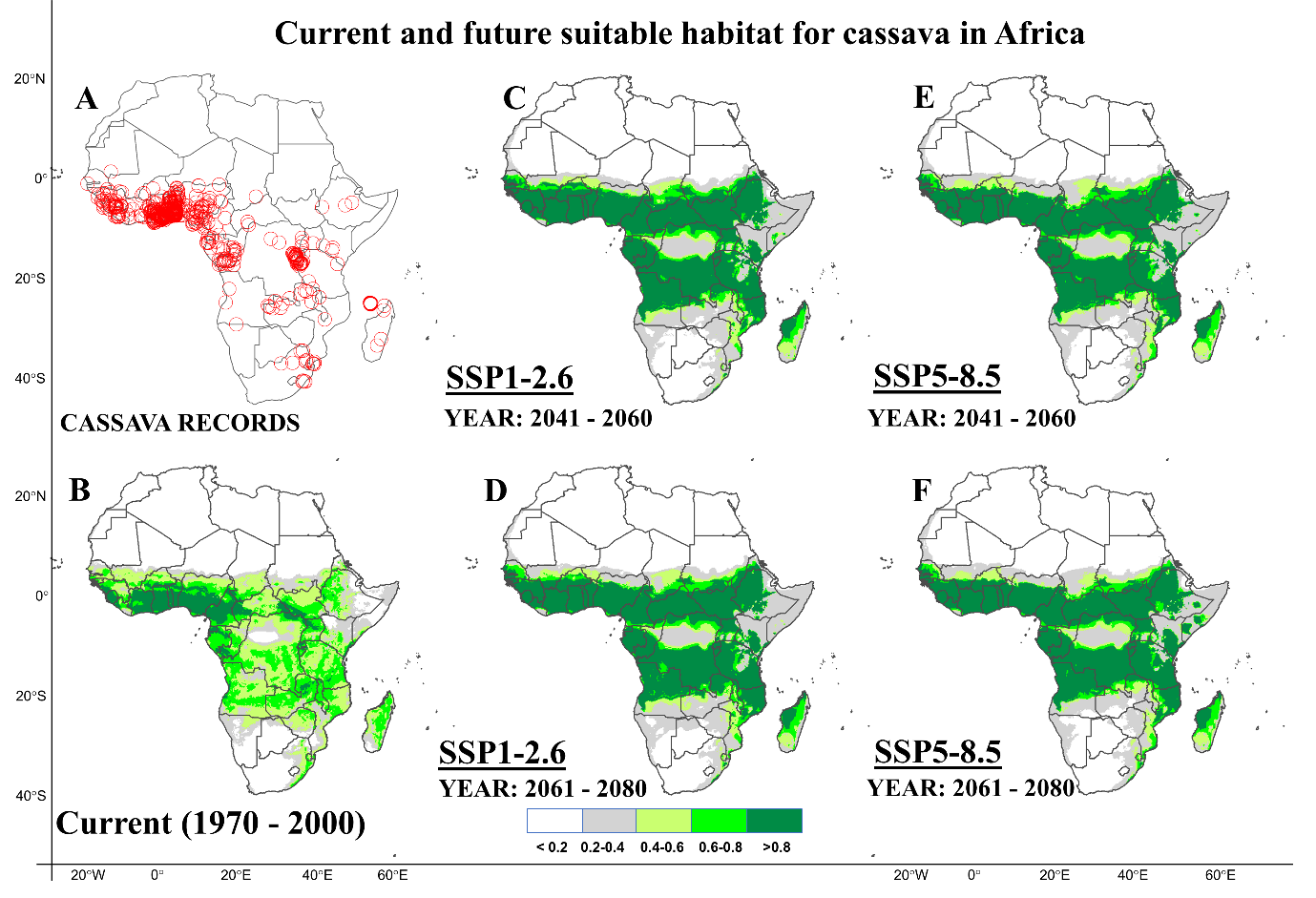


**Figure C3:** Current and future suitable habitats for cassava in Africa. Maps showing A) geolocations of cassava occurrence records provided by the global biodiversity information facility (GIBF; https://www.gbif.org), B) predicted spatial distribution of cassava under historical climate conditions (1970 - 2000). Future prediction of the suitable habitat under two shared social-economic pathways C) SSP1-2.6 (2050s), D) SSP1-2.6 (2070s), D) SSP5-8.5 and E) SSP5-8.5 using the **MIROC-ES2L** model.


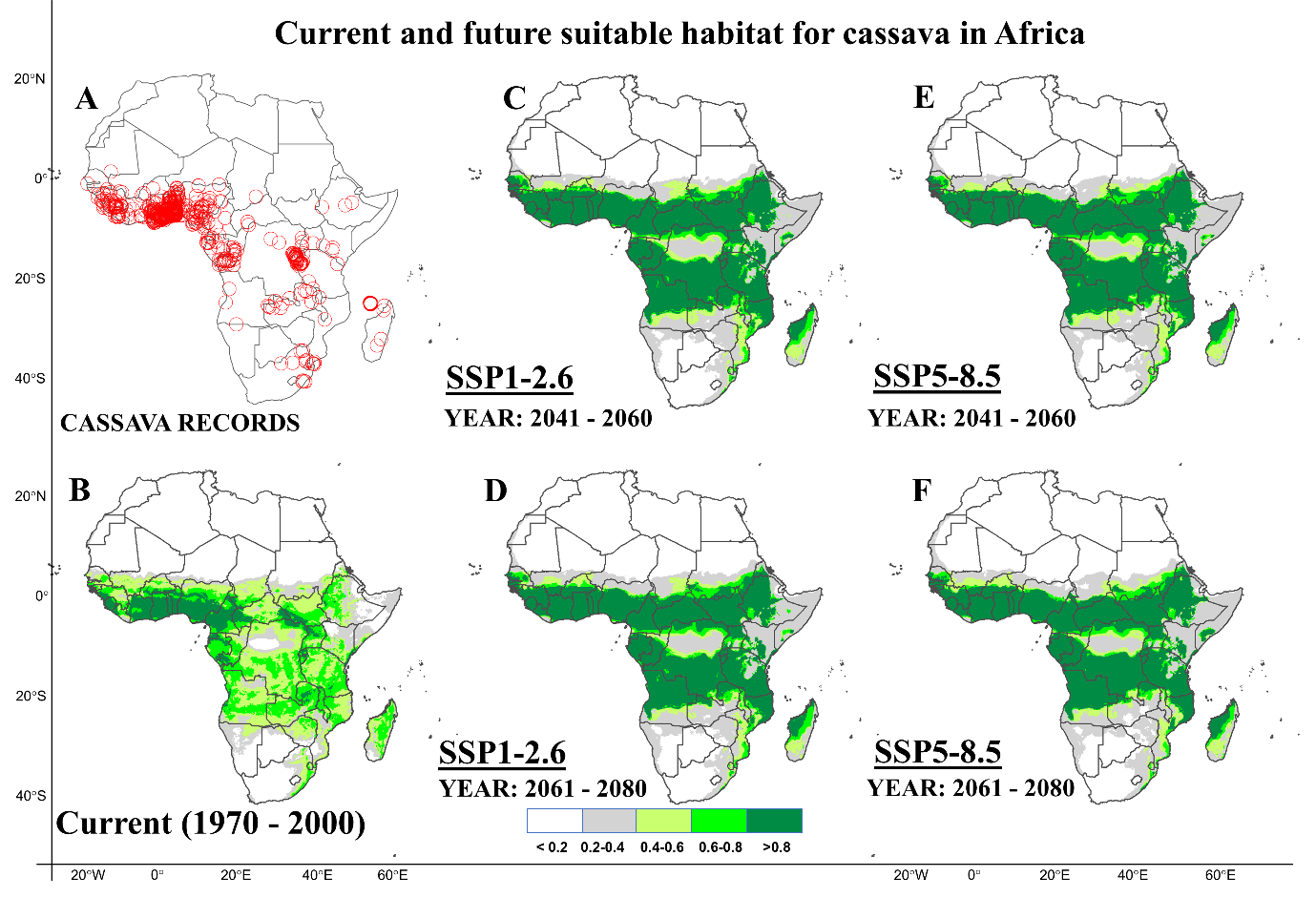


**Figure C4:** Current and future suitable habitats for cassava in Africa. Maps showing A) geolocations of cassava occurrence records provided by the global biodiversity information facility (GIBF; https://www.gbif.org), B) predicted spatial distribution of cassava under historical climate conditions (1970 - 2000). Future prediction of the suitable habitat under two shared social-economic pathways C) SSP1-2.6 (2050s), D) SSP1-2.6 (2070s), D) SSP5-8.5 and E) SSP5-8.5 using the **MIROC6** model.


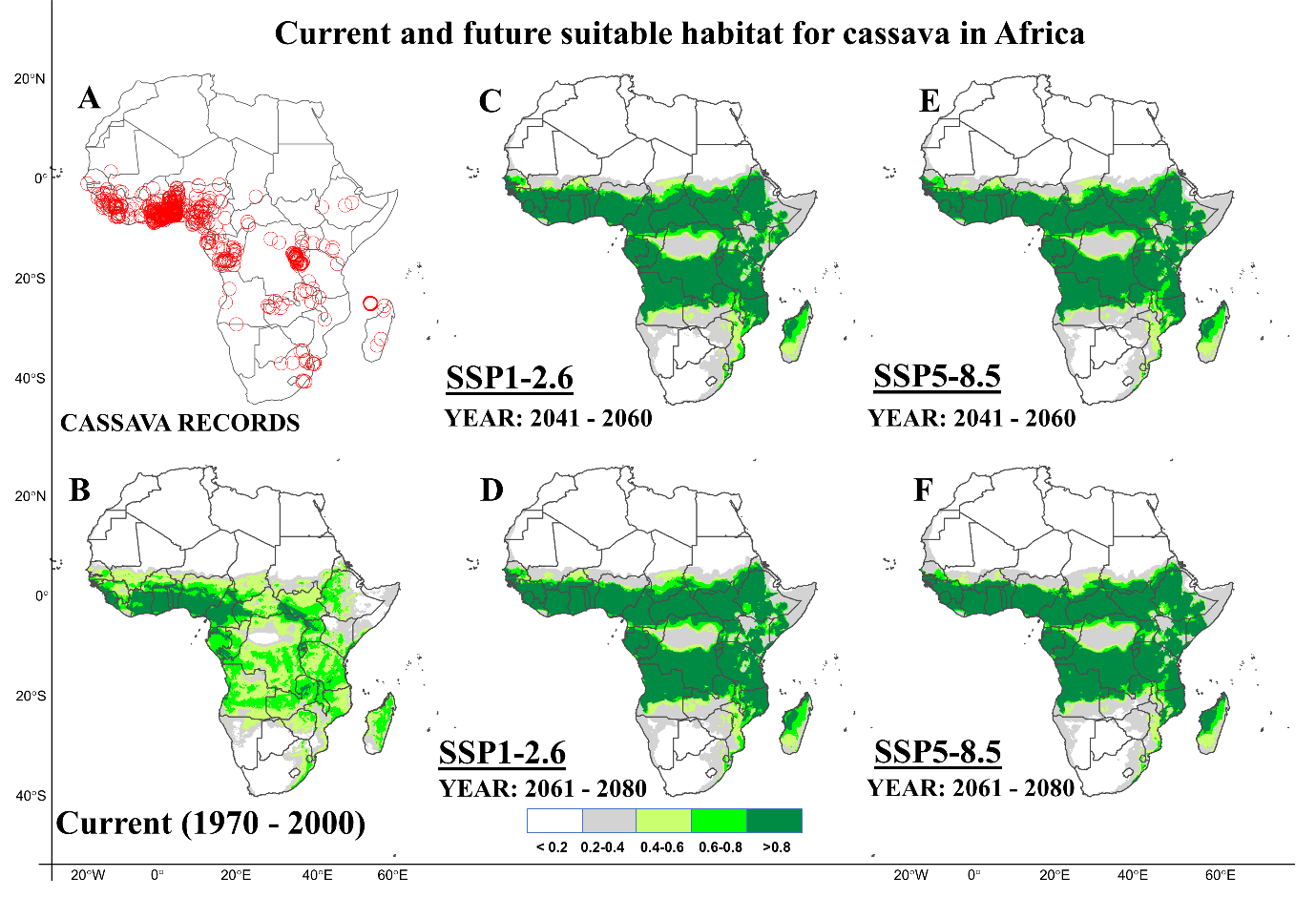


**Figure C5:** Current and future suitable habitats for cassava in Africa. Maps showing A) geolocations of cassava occurrence records provided by the global biodiversity information facility (GIBF; https://www.gbif.org), B) predicted spatial distribution of cassava under historical climate conditions (1970 - 2000). Future prediction of the suitable habitat under two shared social-economic pathways C) SSP1-2.6 (2050s), D) SSP1-2.6 (2070s), D) SSP5-8.5 and E) SSP5-8.5 using the **IPSL-CM6A-LR** model.


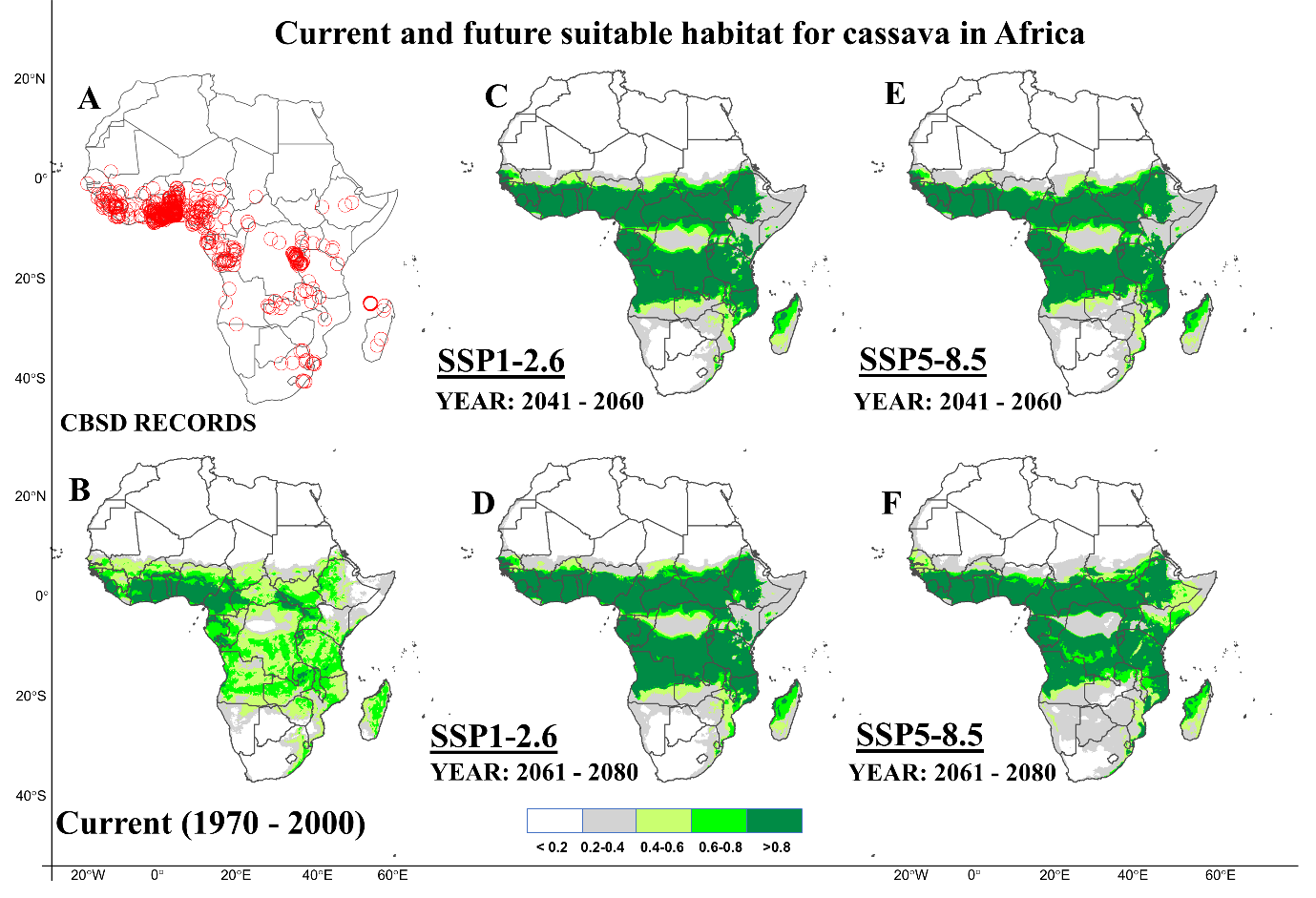


**Figure C6:** Current and future suitable habitats for cassava in Africa. Maps showing A) geolocations of cassava occurrence records provided by the global biodiversity information facility (GIBF; https://www.gbif.org), B) predicted spatial distribution of cassava under historical climate conditions (1970 - 2000). Future prediction of the suitable habitat under two shared social-economic pathways C) SSP1-2.6 (2050s), D) SSP1-2.6 (2070s), D) SSP5-8.5 and E) SSP5-8.5 using the **CNRM-ESM2-1** model.


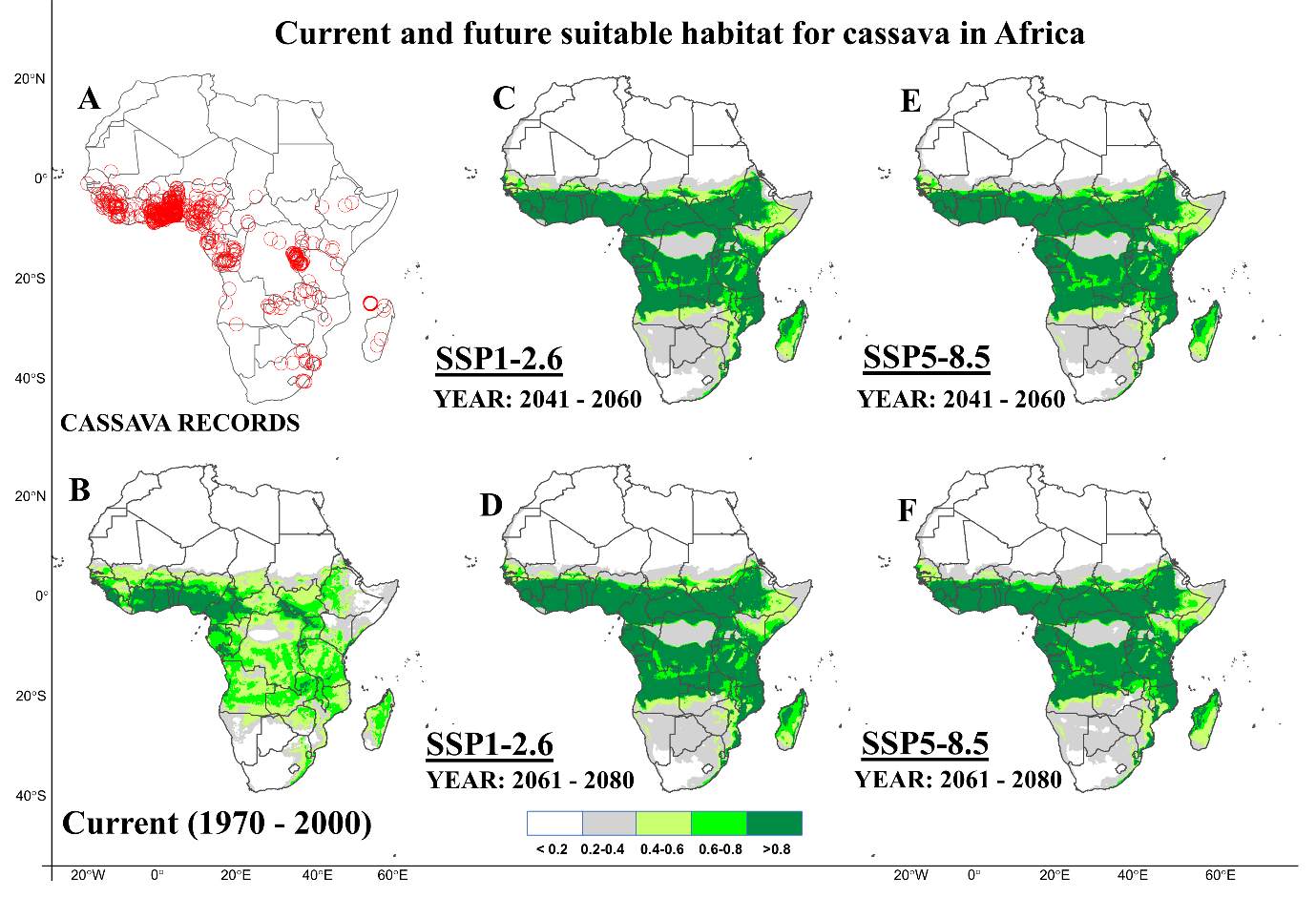


**Figure C7:** Current and future suitable habitats for cassava in Africa. Maps showing A) geolocations of cassava occurrence records provided by the global biodiversity information facility (GIBF; https://www.gbif.org), B) predicted spatial distribution of cassava under historical climate conditions (1970 - 2000). Future prediction of the suitable habitat under two shared social-economic pathways C) SSP1-2.6 (2050s), D) SSP1-2.6 (2070s), D) SSP5-8.5 and E) SSP5-8.5 using the **CNRM-CM6-1** model.


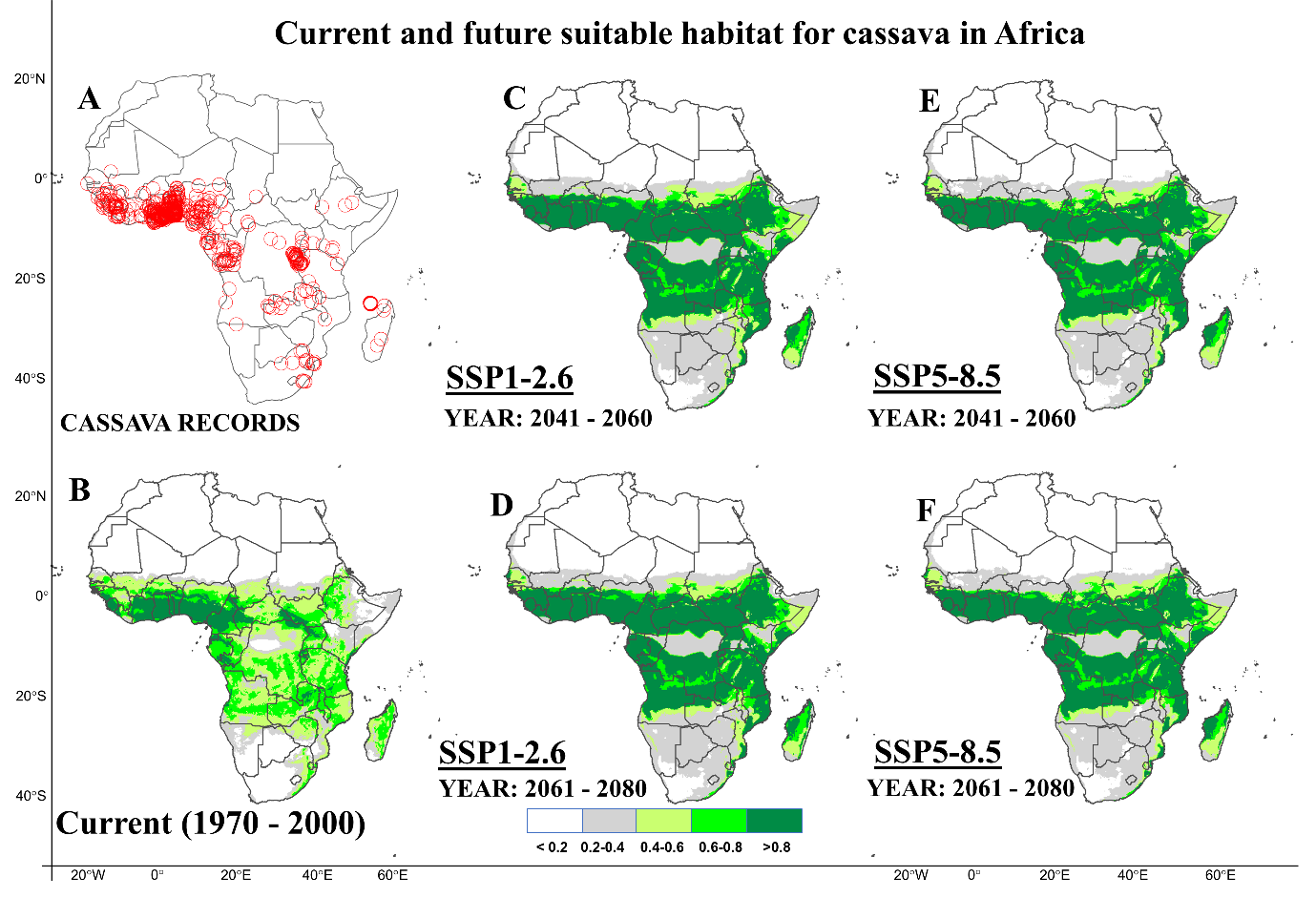


**Figure C8:** Current and future suitable habitats for cassava in Africa. Maps showing A) geolocations of cassava occurrence records provided by the global biodiversity information facility (GIBF; https://www.gbif.org), B) predicted spatial distribution of cassava under historical climate conditions (1970 - 2000). Future prediction of the suitable habitat under two shared social-economic pathways C) SSP1-2.6 (2050s), D) SSP1-2.6 (2070s), D) SSP5-8.5 and E) SSP5-8.5 using the **CanESM5** model.

**Appendix D:** Projection of the current and future habitats at risk of whitefly, Bemisia tabaci invasion in Africa using eight GCM models.


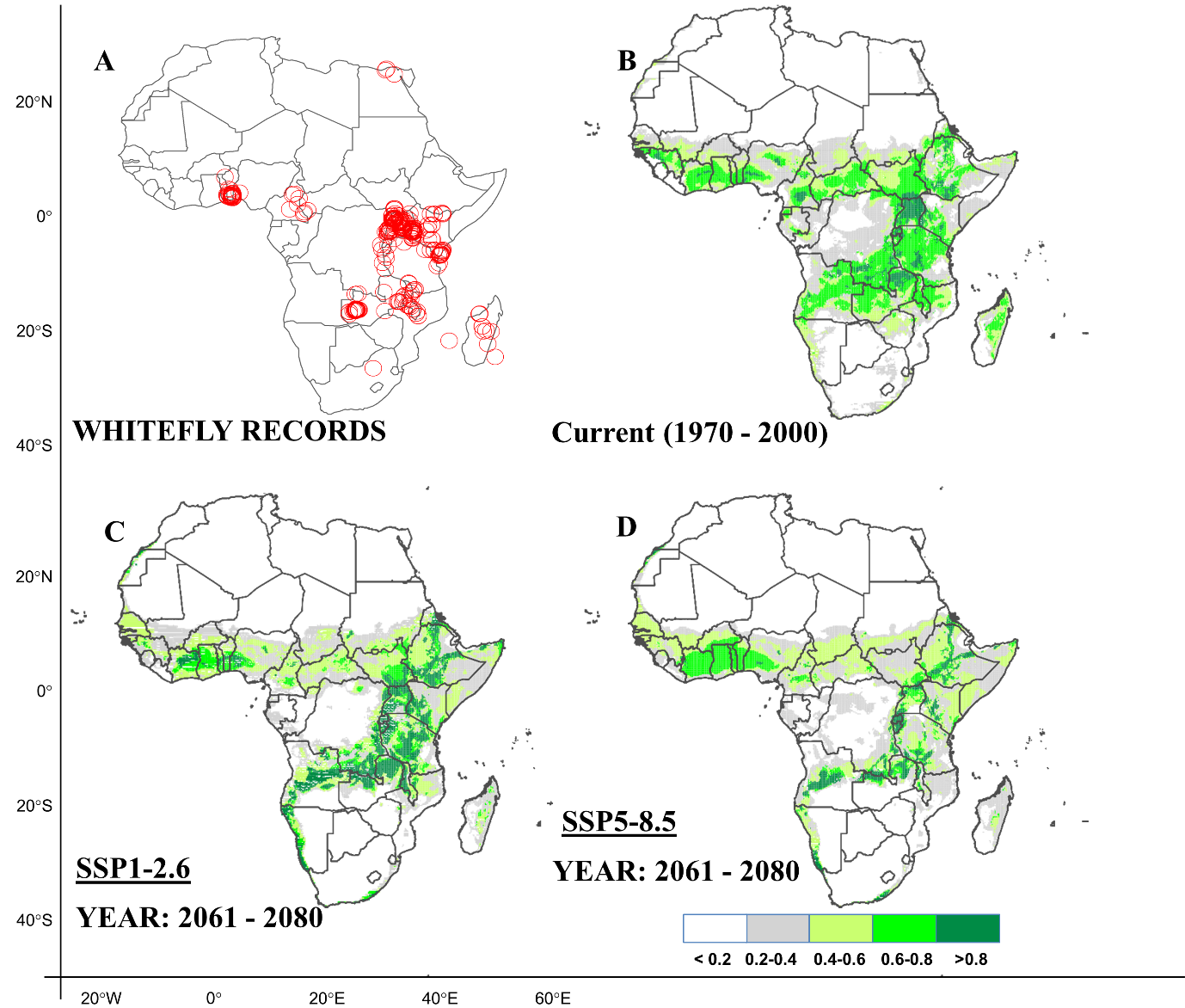


**Figure D1:** Current and future suitable habitats for whitefly in Africa. Plots showing A) geolocations of whitefly occurrence records provided by the global biodiversity information facility (GIBF; <https://www.gbif.org>) and B) the predicted distribution for whitefly under the current climate (1970-2000). Predicted future suitable habitats for whitefly under C) the SSP1-2.6 scenario and D) the SSP5-8.5 scenario, using the **BCC-CSM2-MR** model for the year 2070s. The figures for the SSP1-2.6 and SSP5-8.5 scenarios for the year 2050s are presented in **Fig. 3** of the main text.

**
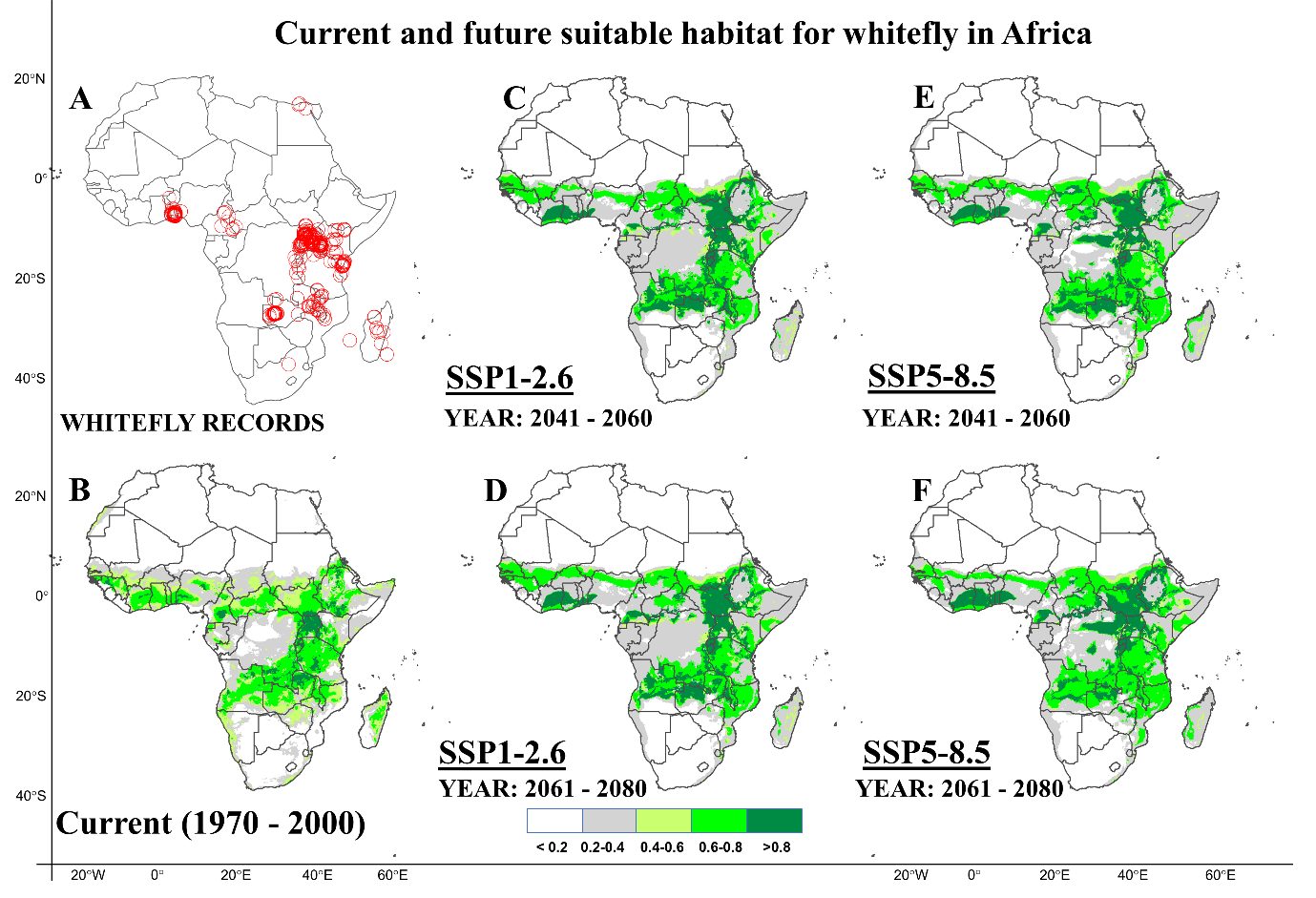
**

**Figure D2:** Current and future suitable habitats for whitefly in Africa. Maps showing A) geolocations of whitefly, *B. tabaci* occurrence records provided by the global biodiversity information facility (GIBF; https://www.gbif.org), B) predicted spatial distribution for whitefly under historical climate conditions. Future prediction of suitable habitats for whitefly under two shared social-economic pathways C) SSP1-2.6 (2050s), D) SSP1-2.6 (2070s), E) SSP5-8.5 (2050s) and F) SSP5-8.5 (2070s) using the **MRI-ESM2-0** model.

**
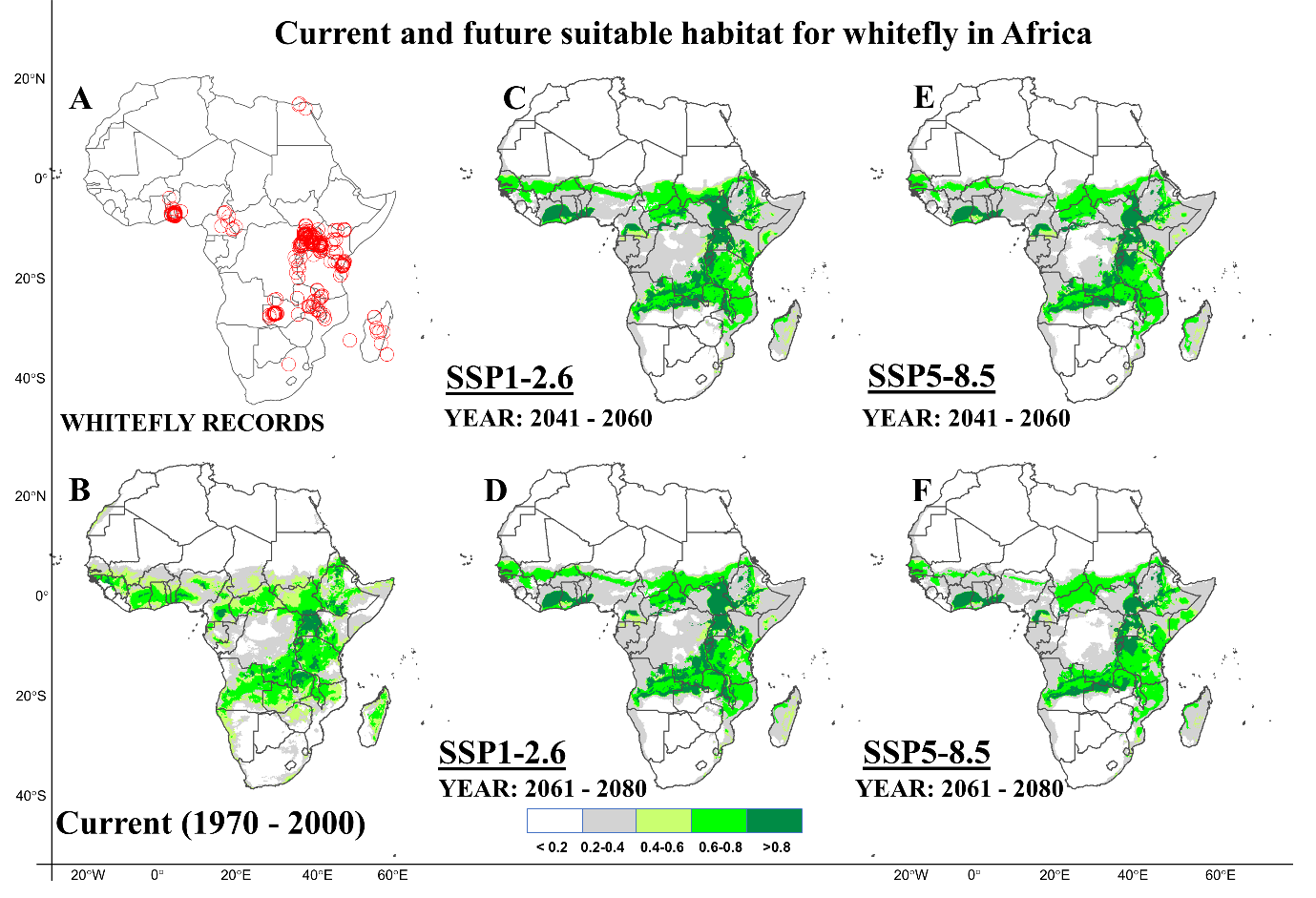
**

**Figure D3:** Current and future suitable habitats for whitefly in Africa. Maps showing A) geolocations of whitefly, *B. tabaci* occurrence records provided by the global biodiversity information facility (GIBF; https://www.gbif.org), B) predicted spatial distribution for whitefly under historical climate conditions. Future prediction of suitable habitats for whitefly under two shared social-economic pathways C) SSP1-2.6 (2050s), D) SSP1-2.6 (2070s), E) SSP5-8.5 (2050s) and F) SSP5-8.5 (2070s) using the **MIROC-ES2L** model.


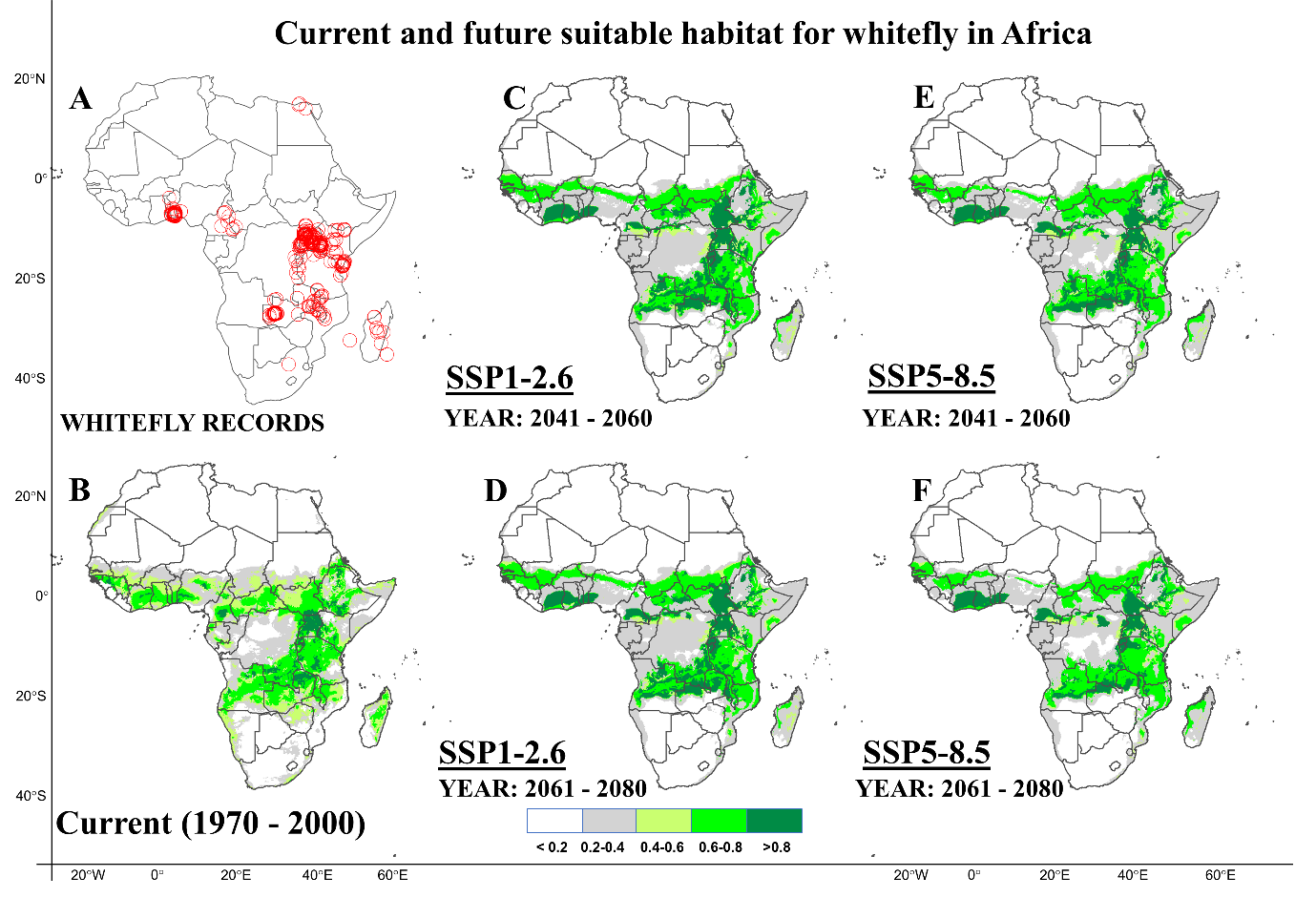


**Figure D4:** Current and future suitable habitats for whitefly in Africa. Maps showing A) geolocations of whitefly, *B. tabaci* occurrence records provided by the global biodiversity information facility (GIBF; https://www.gbif.org), B) predicted spatial distribution for whitefly under historical climate conditions. Future prediction of suitable habitats for whitefly under two shared social-economic pathways C) SSP1-2.6 (2050s), D) SSP1-2.6 (2070s), E) SSP5-8.5 (2050s) and F) SSP5-8.5 (2070s) using the **MIROC6** model.


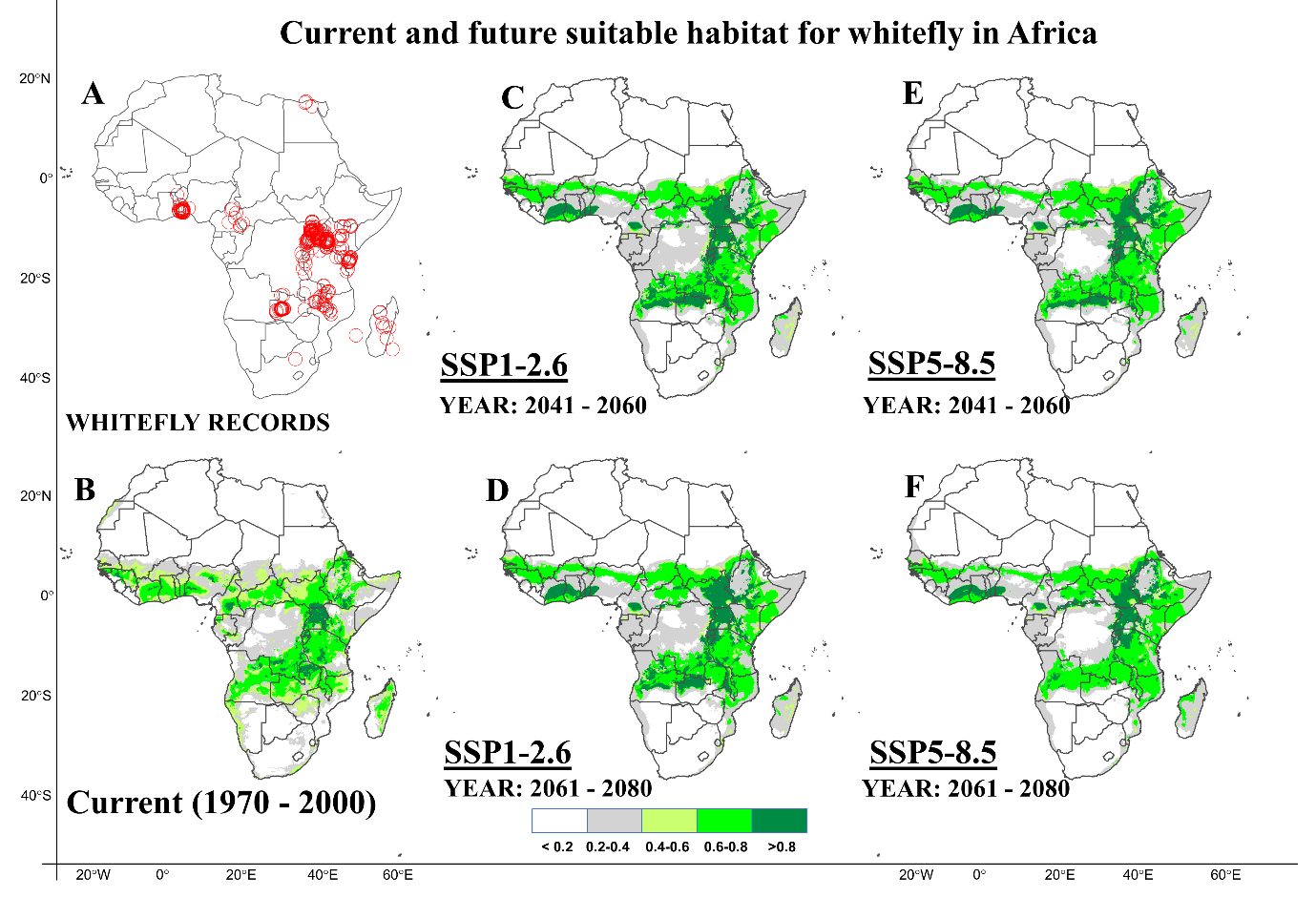


**Figure D5:** Current and future suitable habitats for whitefly in Africa. Maps showing A) geolocations of whitefly, *B. tabaci* occurrence records provided by the global biodiversity information facility (GIBF; https://www.gbif.org), B) predicted spatial distribution for whitefly under historical climate conditions. Future prediction of suitable habitats for whitefly under two shared social-economic pathways C) SSP1-2.6 (2050s), D) SSP1-2.6 (2070s), E) SSP5-8.5 (2050s) and F) SSP5-8.5 (2070s) using the **IPSL-CM6A-LR** model.


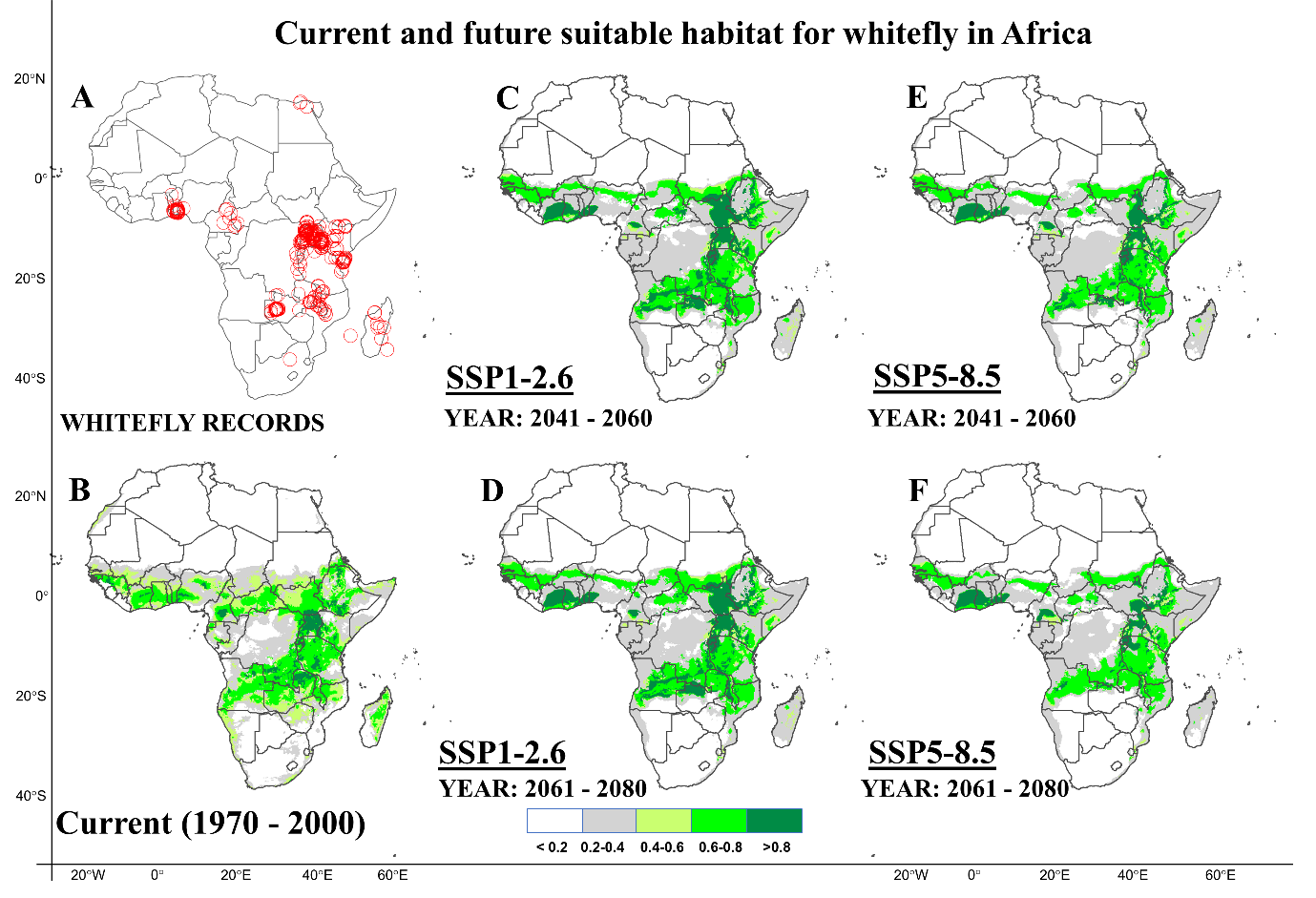


**Figure D6:** Current and future suitable habitats for whitefly in Africa. Maps showing A) geolocations of whitefly, *B. tabaci* occurrence records provided by the global biodiversity information facility (GIBF; https://www.gbif.org), B) predicted spatial distribution for whitefly under historical climate conditions. Future prediction of suitable habitats for whitefly under two shared social-economic pathways C) SSP1-2.6 (2050s), D) SSP1-2.6 (2070s), E) SSP5-8.5 (2050s) and F) SSP5-8.5 (2070s) using the **CNRM-ESM2-1** model.


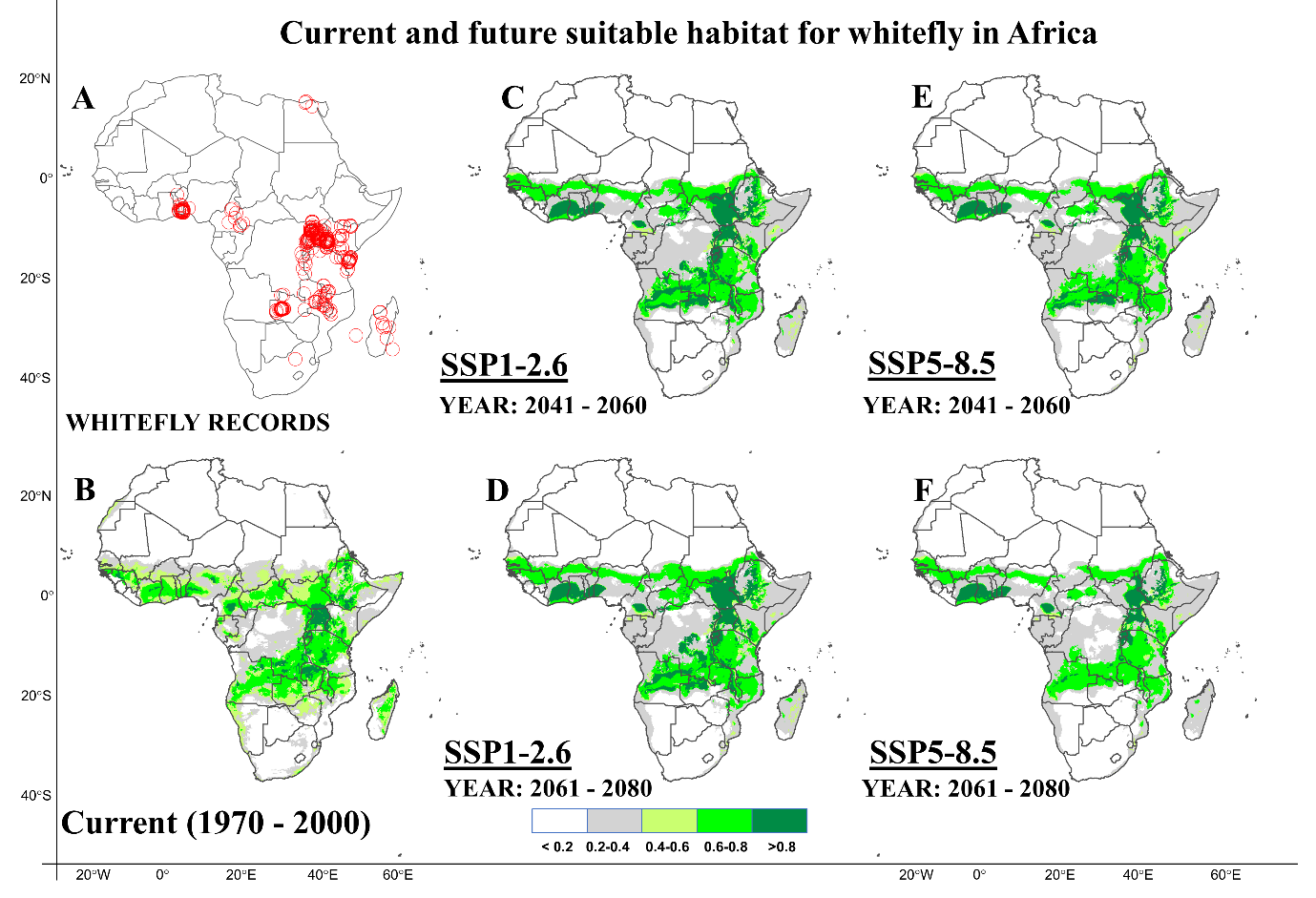


**Figure D7.** Current and future suitable habitats for whitefly in Africa. Maps showing A) geolocations of whitefly, *B. tabaci* occurrence records provided by the global biodiversity information facility (GIBF; https://www.gbif.org), B) predicted spatial distribution for whitefly under historical climate conditions. Future prediction of suitable habitats for whitefly under two shared social-economic pathways C) SSP1-2.6 (2050s), D) SSP1-2.6 (2070s), E) SSP5-8.5 (2050s) and F) SSP5-8.5 (2070s) using the **CNRM-CM6-1** model.


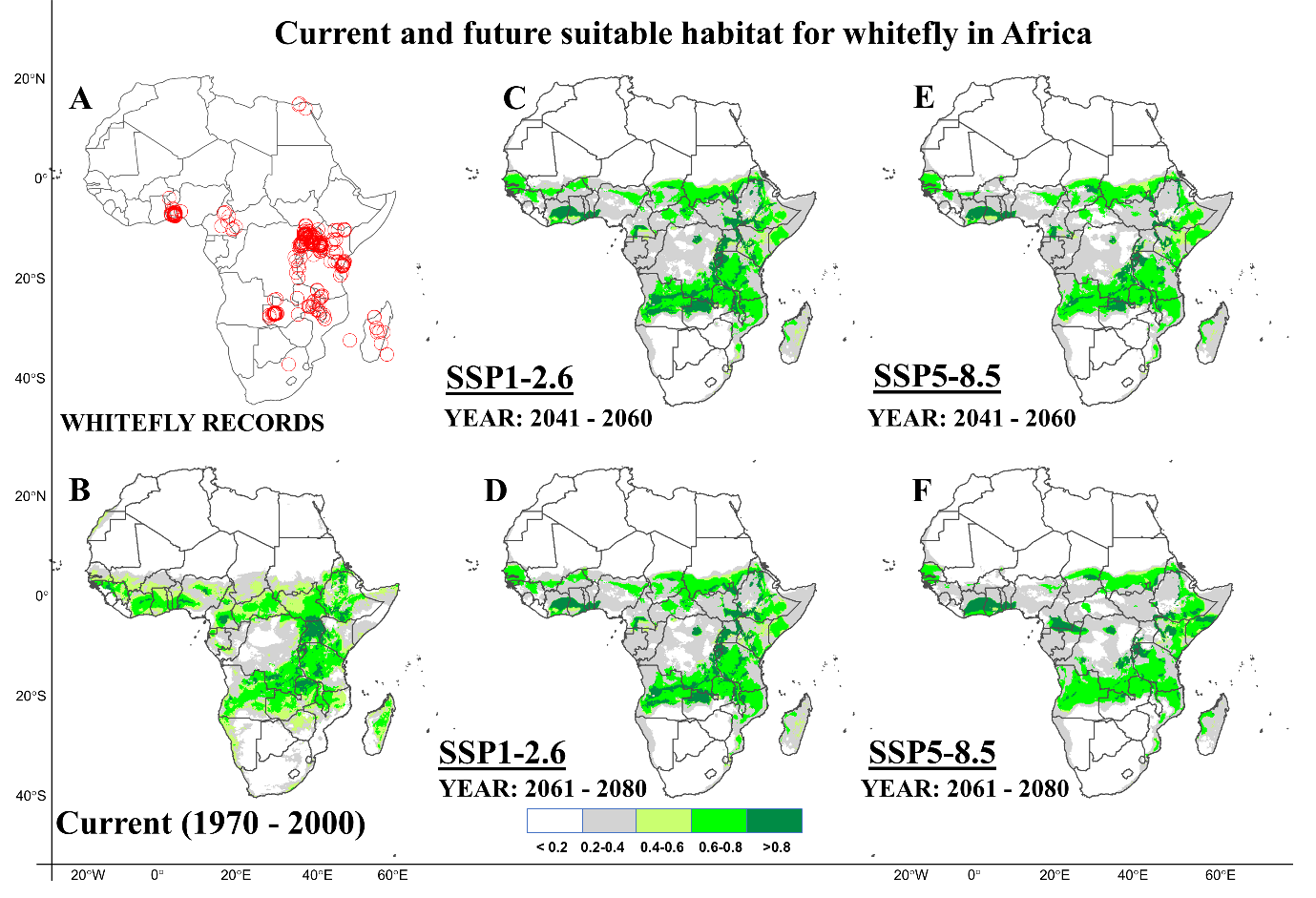


**Figure D8.** Current and future suitable habitats for whitefly in Africa. Maps showing A) geolocations of whitefly, *B. tabaci* occurrence records provided by the global biodiversity information facility (GIBF; https://www.gbif.org), B) predicted spatial distribution for whitefly under historical climate conditions. Future prediction of suitable habitats for whitefly under two shared social-economic pathways C) SSP1-2.6 (2050s), D) SSP1-2.6 (2070s), E) SSP5-8.5 (2050s) and F) SSP5-8.5 (2070s) using the **CanESM5** model.

**Appendix E:** Projection of the current and future habitats at risk of cassava brown streak invasion in Africa using eight GCM models


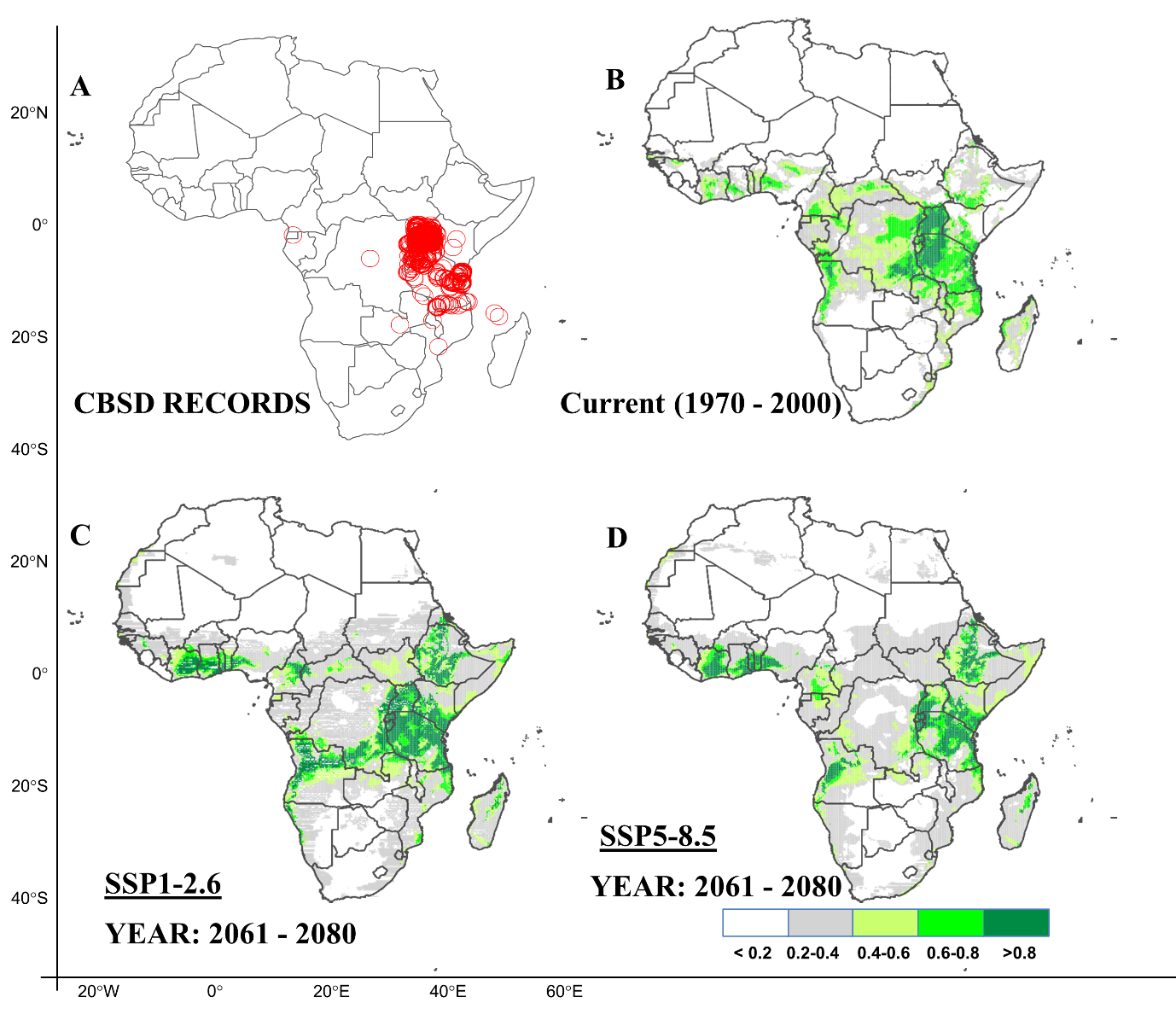


**Figure E1:** Current and future suitable habitats for CBSD in Africa. Plots showing A) geolocations of whitefly occurrence records provided by the global biodiversity information facility (GIBF; <https://www.gbif.org>) and personal correspondence with cassava diagnostic project leader in East Africa B) the predicted distribution for CBSD under the current climate (1970-2000). Predicted future suitable habitats for CBSD under C) the SSP1-2.6 scenario and D) the SSP5-8.5 scenario, using the **BCC-CSM2-MR** model for the year 2070s. The figures the SSP1-2.6 and SSP5-8.5 scenarios for the year 2050s are presented in **Fig. 4** of the main text.


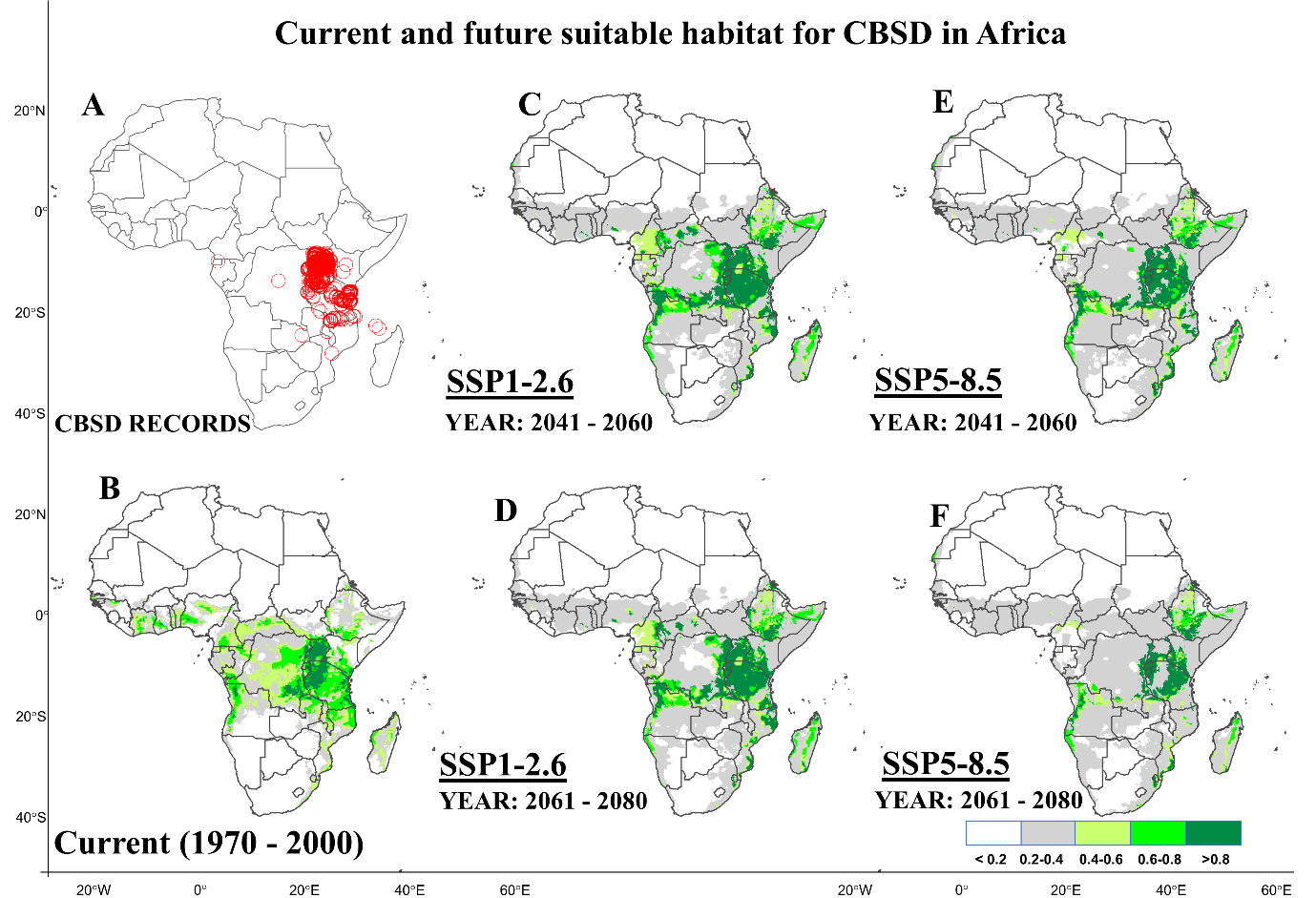


**Figure E2.** Current and future suitable habitats for CBSD in Africa. Plots showing A) geolocations of cassava brown streak disease (CBSD) occurrence records provided by the global biodiversity information facility (GIBF; https://www.gbif.org) and personal correspondence with cassava diagnostic project leader in East Africa B) predicted current distribution for whitefly. Future prediction of suitable habitats for whitefly under two shared social-economic pathways C) SSP1-2.6 (2050s), D) SSP1-2.6 (2070s), E) SSP5-8.5 (2050s) and F) SSP5-8.5 (2070s) using the **MRI-ESM2-0** model.

**
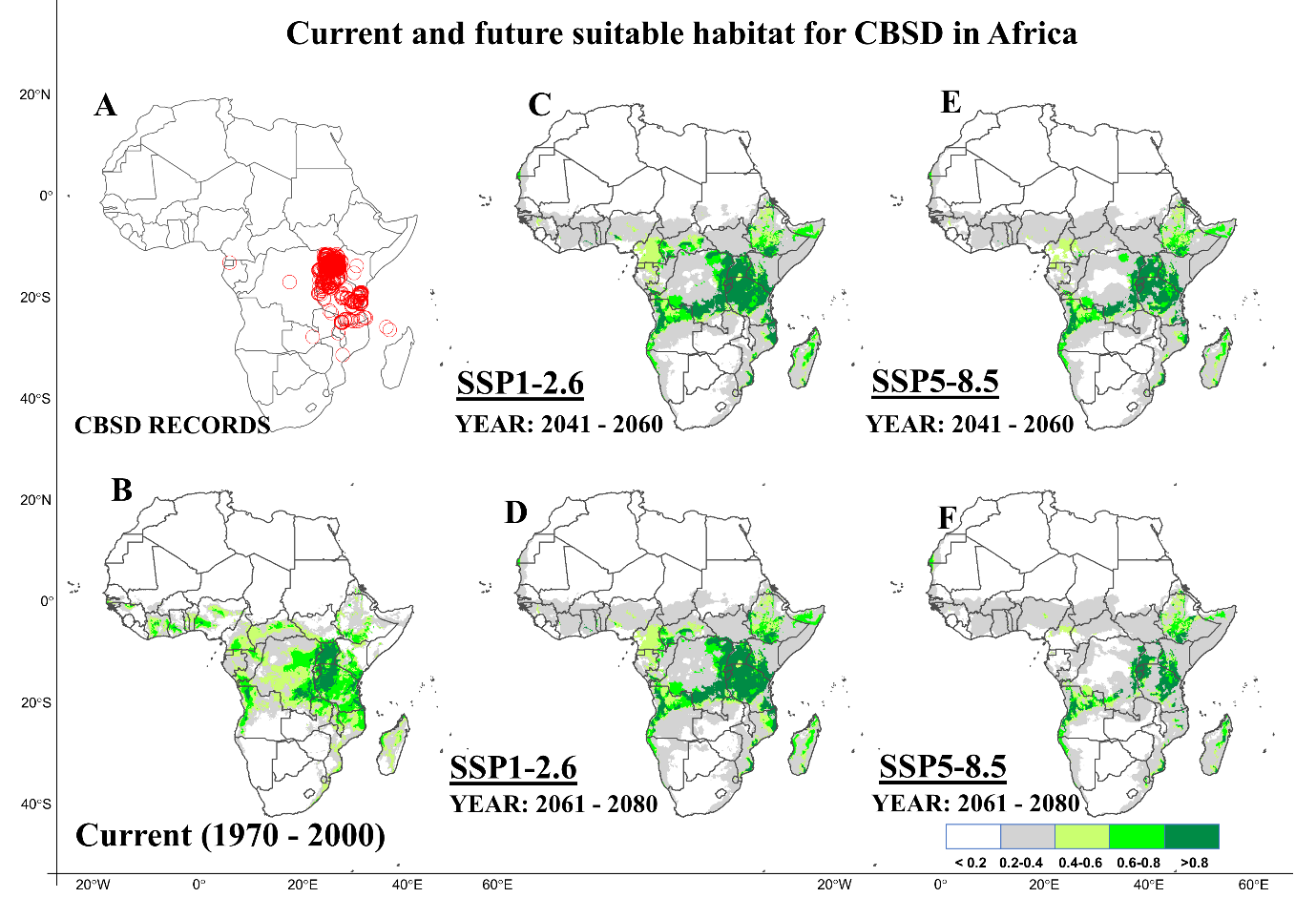
**

**Figure E3.** Current and future suitable habitats for CBSD in Africa. Plots showing A) geolocations of cassava brown streak disease occurrence records provided by the global biodiversity information facility (GIBF; https://www.gbif.org) and personal correspondence with cassava diagnostic project leader in East Africa B) predicted current distribution for whitefly. Future prediction of suitable habitats for whitefly under two shared social-economic pathways C) SSP1-2.6 (2050s), D) SSP1-2.6 (2070s), E) SSP5-8.5 (2050s) and F) SSP5-8.5 (2070s) using the **MIROC-ES2L** model.


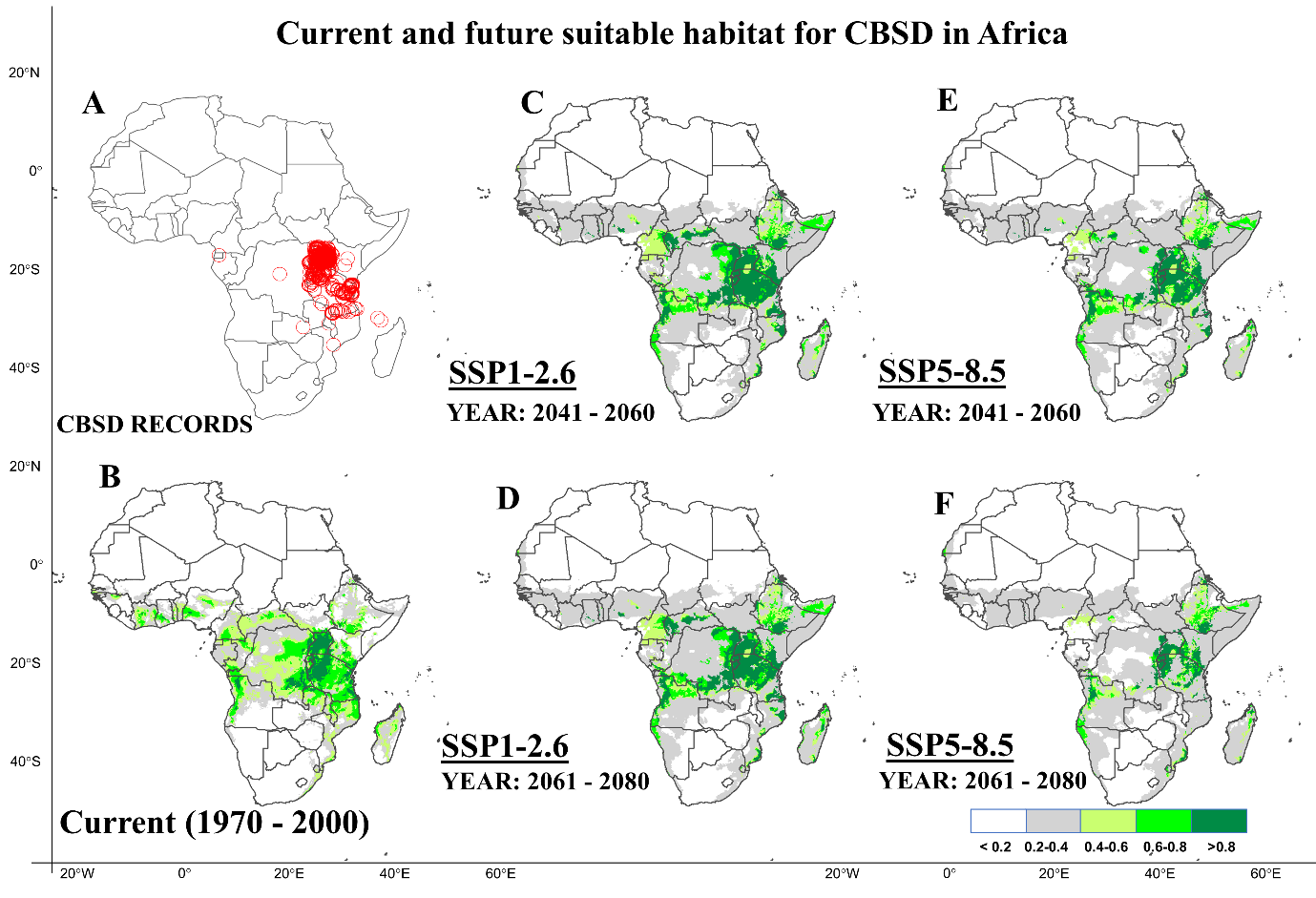


**Figure E4.** Current and future suitable habitats for CBSD in Africa. Plots showing A) geolocations of cassava brown streak disease occurrence records provided by the global biodiversity information facility (GIBF; https://www.gbif.org) and personal correspondence with cassava diagnostic project leader in East Africa B) predicted current distribution for whitefly. Future prediction of suitable habitats for whitefly under two shared social-economic pathways C) SSP1-2.6 (2050s), D) SSP1-2.6 (2070s), E) SSP5-8.5 (2050s) and F) SSP5-8.5 (2070s) using the **MIROC6** model.

**
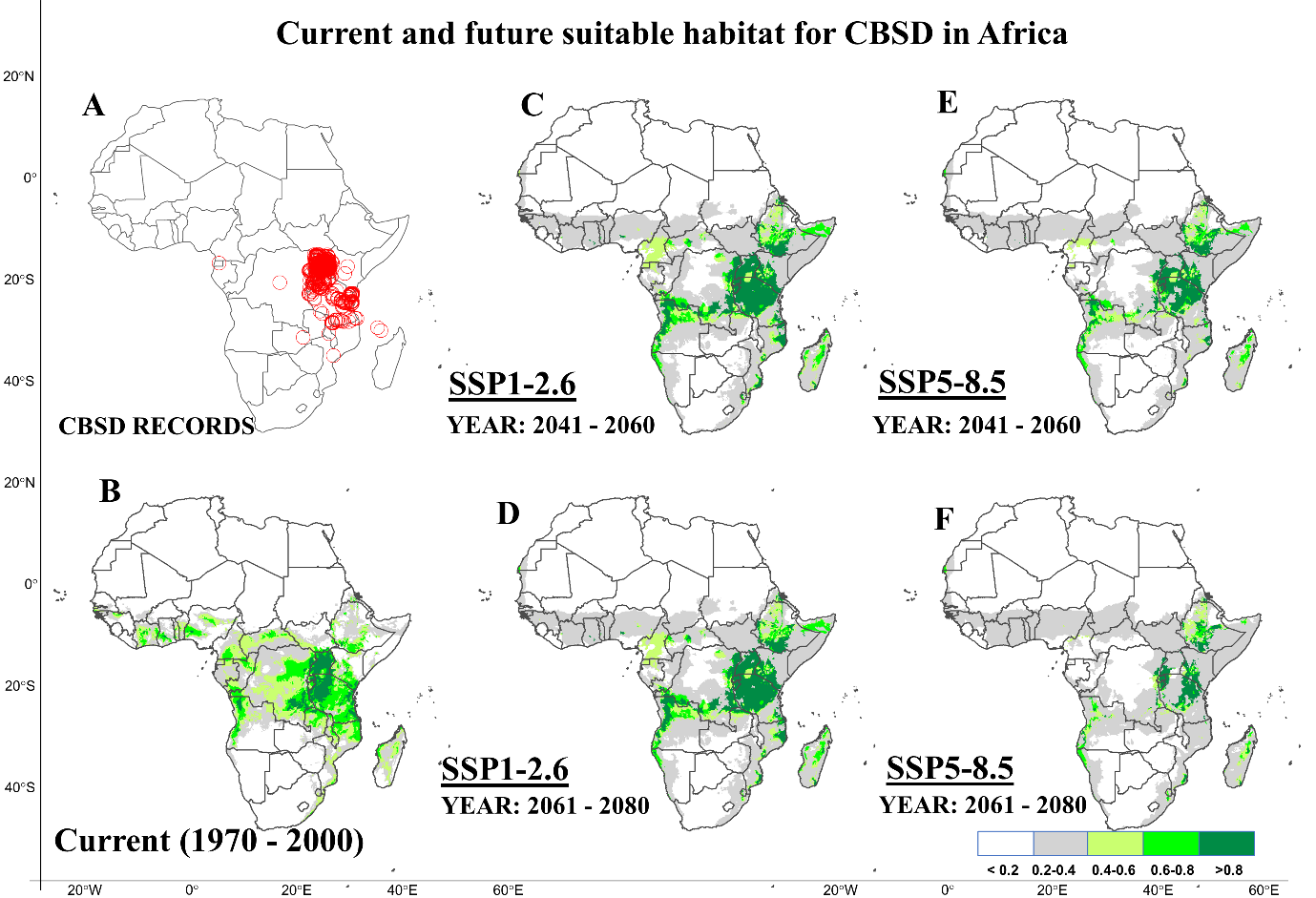
**

**Figure E5.** Current and future suitable habitats for CBSD in Africa. Plots showing A) geolocations of cassava brown streak disease occurrence records provided by the global biodiversity information facility (GIBF; https://www.gbif.org) and personal correspondence with cassava diagnostic project leader in East Africa B) predicted current distribution for whitefly. Future prediction of suitable habitats for whitefly under two shared social-economic pathways C) SSP1-2.6 (2050s), D) SSP1-2.6 (2070s), E) SSP5-8.5 (2050s) and F) SSP5-8.5 (2070s) using the **IPSL-CM6A-LR** model.


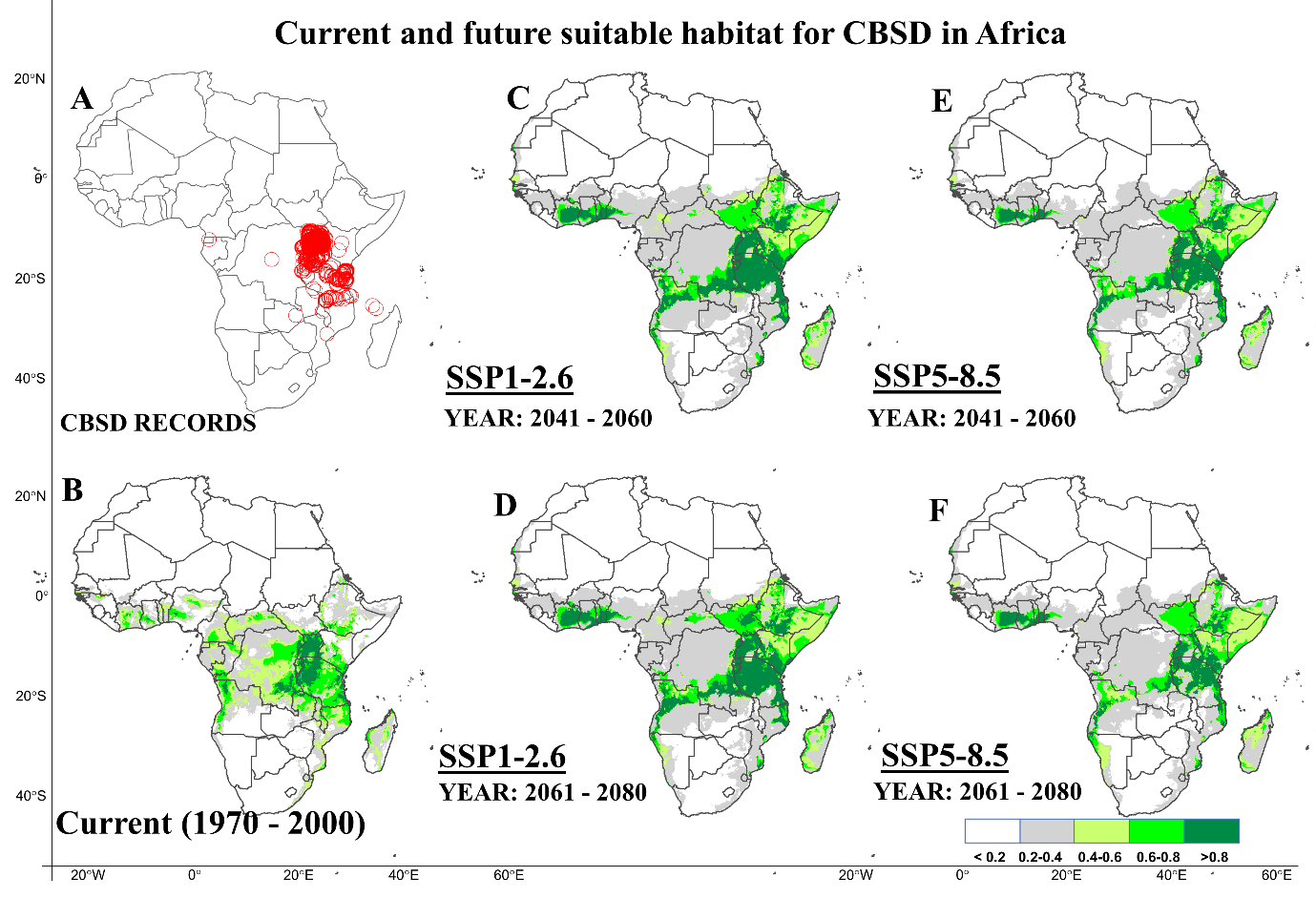


**Figure E6.** Current and future suitable habitats for CBSD in Africa. Plots showing A) geolocations of cassava brown streak disease occurrence records provided by the global biodiversity information facility (GIBF; https://www.gbif.org) and personal correspondence with cassava diagnostic project leader in East Africa B) predicted current distribution for whitefly. Future prediction of suitable habitats for whitefly under two shared social-economic pathways C) SSP1-2.6 (2050s), D) SSP1-2.6 (2070s), E) SSP5-8.5 (2050s) and F) SSP5-8.5 (2070s) using the **CNRM-ESM2-1** model.


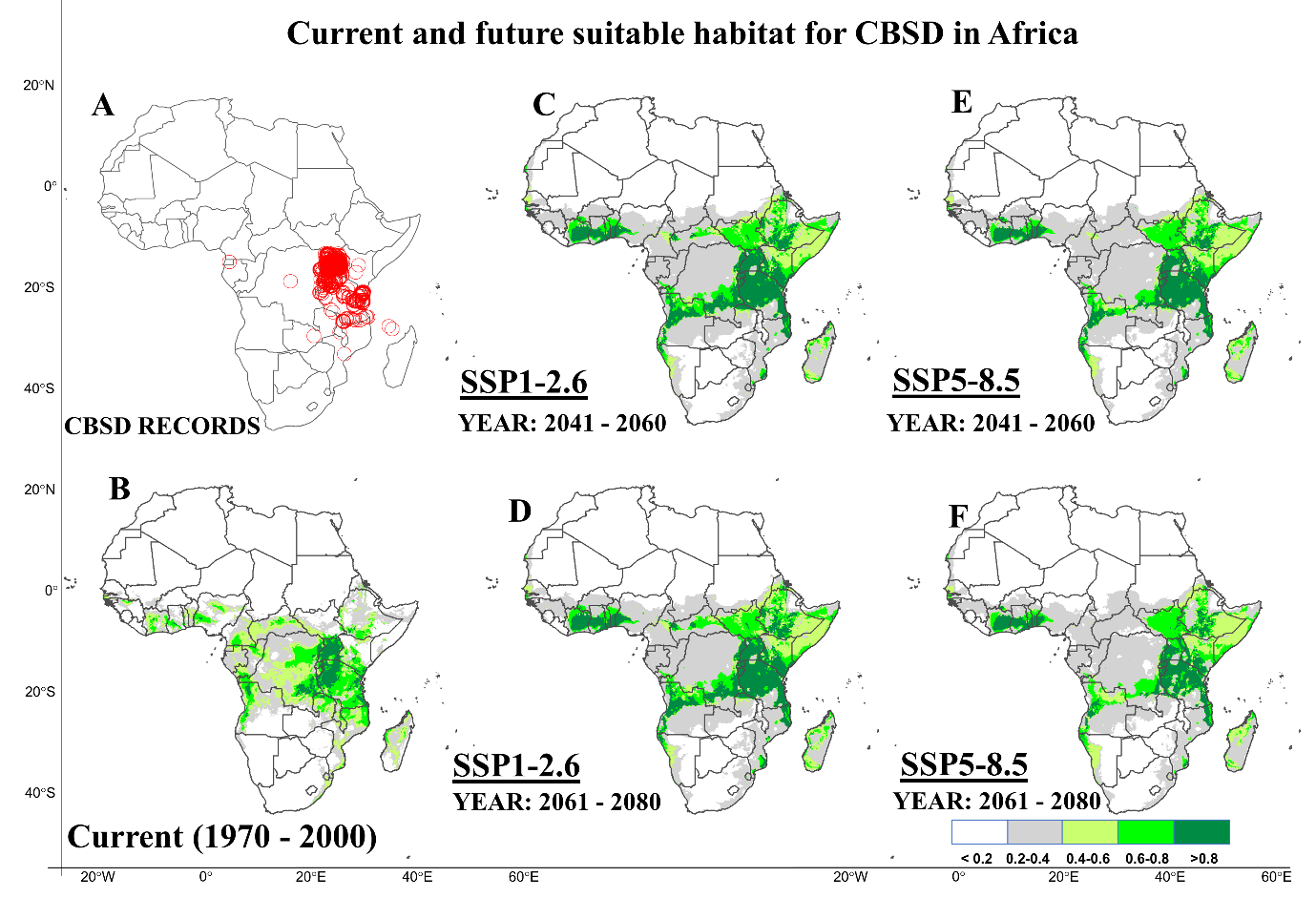


**Figure E7.** Current and future suitable habitats for CBSD in Africa. Plots showing A) geolocations of cassava brown streak disease occurrence records provided by the global biodiversity information facility (GIBF; https://www.gbif.org) and personal correspondence with cassava diagnostic project leader in East Africa B) predicted current distribution for whitefly. Future prediction of suitable habitats for whitefly under two shared social-economic pathways C) SSP1-2.6 (2050s), D) SSP1-2.6 (2070s), E) SSP5-8.5 (2050s) and F) SSP5-8.5 (2070s) using the **CNRM-CM6-1** model.

**Appendix F:** Impact of species lumping in predicting the current and future distribution of *Bemisia tabaci* species complex in Africa


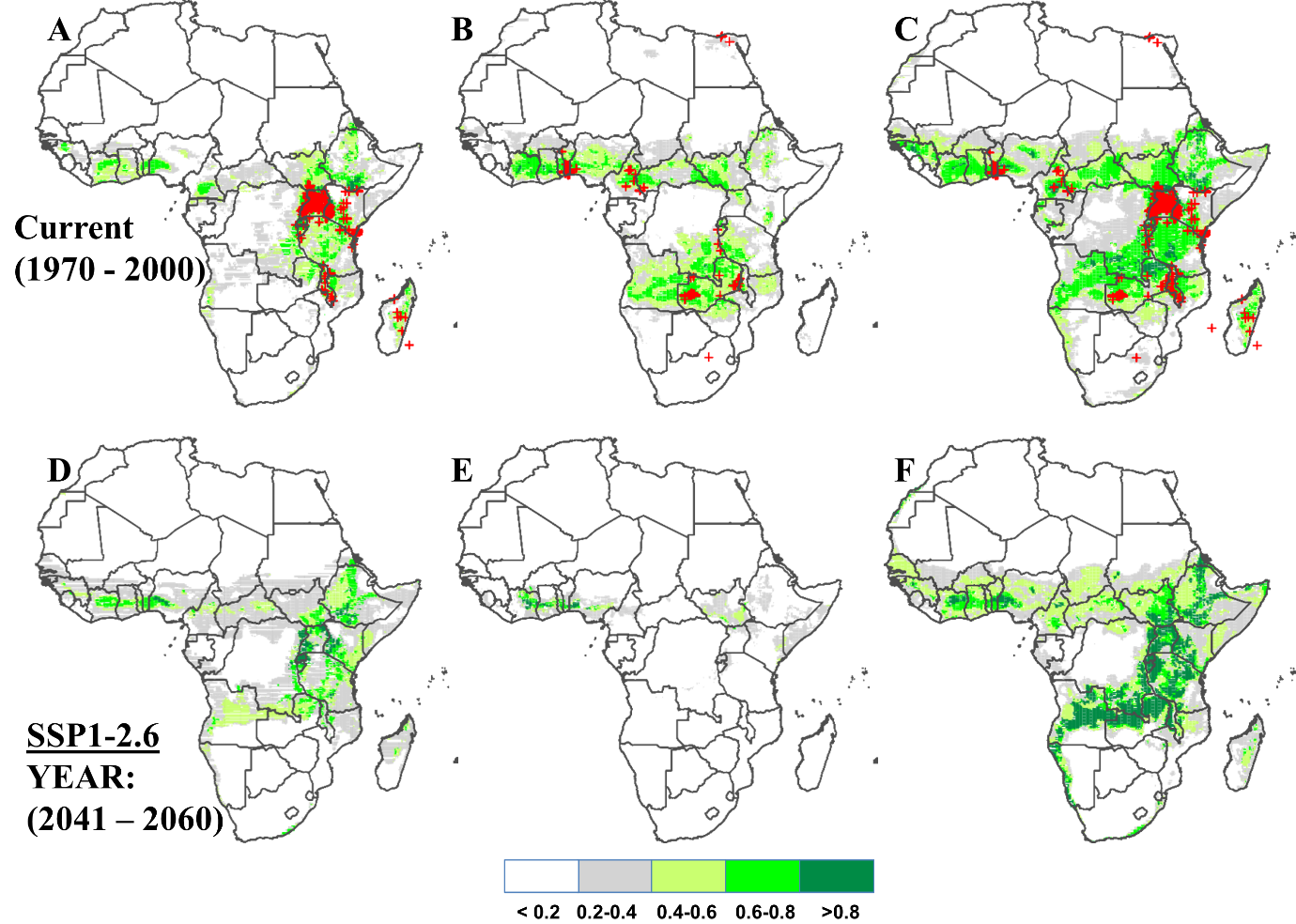


**Figure F1.** Current and future suitable habitats for members of *Bemisia tabaci* species in Africa. (A, D) Predicted current and future distribution for *Bemisia tabaci* species (SSA1) overlayed with geolocations from East Africa under shared social-economic pathways (SSP1-2.6) using the **BCC-CSM2-MR** model for the year 2050. (B, E) Predicted current and future distribution for *Bemisia tabaci* species (SSA2-5) overlayed with geolocations from Central and West Africa under shared social-economic pathways (SSP1-2.6) using the **BCC-CSM2-MR** model for the year 2050 and (C, F) Predicted current and future distribution for *Bemisia tabaci* species overlayed with geolocations from entire Africa under shared social-economic pathways (SSP1-2.6) using the **BCC-CSM2-MR** model for the year 2050.

*Comparison of the performance of SDMs on two groups of Bemisia tabaci species*

The ensemble of four models produced on average, an AUC of 98.7% for SSA1 records, 97.6% for *SSA2-5* and 98.3%  for the lumped records of species (SSA1 and SSA2-5).

**Literature cited**

Hajima, T., Watanabe, M., Yamamoto, A., Tatebe, H., Noguchi, M., Abe, M., … Kawamiya, M. (2019). Description of the MIROC-ES2L Earth system model and evaluation of its climate–biogeochemical processes and feedbacks. *Geoscientific Model Development Discussions*, *5*(October), 1–73.

Swart, N. C., Cole, J. N. S., Kharin, V. V., Lazare, M., Scinocca, J. F., Gillett, N. P., … Winter, B. (2019). The Canadian Earth System Model version 5 (CanESM5.0.3). *Geoscientific Model Development*, *12*(11), 4823–4873. https://doi.org/10.5194/gmd-12-4823-2019

Tatebe, H., Ogura, T., Nitta, T., Komuro, Y., Ogochi, K., Takemura, T., … Kimoto, M. (2019). Description and basic evaluation of simulated mean state, internal variability, and climate sensitivity in MIROC6. *Geoscientific Model Development*, *12*(7), 2727–2765. https://doi.org/10.5194/gmd-12-2727-2019

Wu, T., Lu, Y., Fang, Y., Xin, X., Li, L., Li, W., … Liu, X. (2019). The Beijing Climate Center Climate System Model (BCC-CSM): The main progress from CMIP5 to CMIP6. *Geoscientific Model Development*, *12*(4), 1573–1600. https://doi.org/10.5194/gmd-12-1573-2019

Yukimoto, S., Kawai, H., Koshiro, T., Oshima, N., Yoshida, K., Urakawa, S., … Ishii, M. (2019). The meteorological research institute Earth system model version 2.0, MRI-ESM2.0: Description and basic evaluation of the physical component. *Journal of the Meteorological Society of Japan*, *97*(5), 931–965. https://doi.org/10.2151/jmsj.2019-051
